# Multi-omics Analysis Sheds Light on the Evolution and the Intracellular Lifestyle Strategies of Spotted Fever Group *Rickettsia* spp.

**DOI:** 10.3389/fmicb.2017.01363

**Published:** 2017-07-20

**Authors:** Khalid El Karkouri, Malgorzata Kowalczewska, Nicholas Armstrong, Said Azza, Pierre-Edouard Fournier, Didier Raoult

**Affiliations:** Unité de Recherche en Maladies Infectieuses et Tropicales Emergentes, UM63, Centre National De La Recherche Scientifique 7278, IRD 198, Institut National De La Santé Et De La Recherche Médicale U1095, Institut Hospitalo-Universitaire Méditerranée-Infection, Aix-Marseille Université Marseille, France

**Keywords:** *Rickettsia*, intracellular, infectious diseases, virulence, pathogenicity, evolution, genomics, proteomics

## Abstract

Arthropod-borne *Rickettsia* species are obligate intracellular bacteria which are pathogenic for humans. Within this genus, *Rickettsia slovaca* and *Rickettsia conorii* cause frequent and potentially severe infections, whereas *Rickettsia raoultii* and *Rickettsia massiliae* cause rare and milder infections. All four species belong to spotted fever group (SFG) rickettsiae. However, *R. slovaca* and *R. raoultii* cause scalp eschar and neck lymphadenopathy (SENLAT) and are mainly associated with *Dermacentor* ticks, whereas the other two species cause Mediterranean spotted fever (MSF) and are mainly transmitted by *Rhipicephalus* ticks. To identify the potential genes and protein profiles and to understand the evolutionary processes that could, comprehensively, relate to the differences in virulence and pathogenicity observed between these four species, we compared their genomes and proteomes. The virulent and milder agents displayed divergent phylogenomic evolution in two major clades, whereas either SENLAT or MSF disease suggests a discrete convergent evolution of one virulent and one milder agent, despite their distant genetic relatedness. Moreover, the two virulent species underwent strong reductive genomic evolution and protein structural variations, as well as a probable loss of plasmid(s), compared to the two milder species. However, an abundance of mobilome genes was observed only in the less pathogenic species. After infecting *Xenopus laevis* cells, the virulent agents displayed less up-regulated than down-regulated proteins, as well as less number of identified core proteins. Furthermore, their similar and distinct protein profiles did not contain some genes (e.g., *omp*A/B and *rick*A) known to be related to rickettsial adhesion, motility and/or virulence, but may include other putative virulence-, antivirulence-, and/or disease-related proteins. The identified evolutionary forces herein may have a strong impact on intracellular expressions and strategies in these rickettsiae, and that may contribute to the emergence of distinct virulence and diseases in humans. Thus, the current multi-omics data provide new insights into the evolution and fitness of SFG virulence and pathogenicity, and intracellular pathogenic bacteria.

## Introduction

*Rickettsia* species (Order *Rickettsiales*, Family *Rickettsiaceae*) are obligate intracellular bacteria that diverged into three major phylogenetic groups, with arthropod hosts worldwide (Stothard et al., 1994; Raoult and Roux, 1997). This includes the spotted fever group (SFG) associated with ticks, fleas and mites, the typhus group (TG), including *Rickettsia prowazekii* and *Rickettsia typhi* associated with body lice and rat fleas, respectively, and a group containing *Rickettsia bellii* and *R. canadensis*, associated with ticks. Recent studies have divided the SFG group into distinct phylogenetic subgroups (Gillespie et al., 2007; Merhej and Raoult, 2011; Merhej et al., 2014). Moreover, it has been reported that other *Rickettsia* lineages exist, notably associated with amoebas, medusae, ciliates, leeches, or arthropods (Weinert et al., 2009; Merhej and Raoult, 2011; Murray et al., 2016). During their lifecycle, rickettsiae can also infect mammalian hosts, mostly through arthropod bites or feces, causing damage, morbidity, and mortality, as well as a range of mild to severe diseases, such as epidemic typhus and Rocky Mountain spotted fever (RMSF; Parola et al., 2013; Sahni et al., 2013; Portillo et al., 2015). Some *Rickettsia* spp. have been classified as Category B or C bioterrorism pathogens by the National Institute of Allergy and Infectious diseases (NIAID) and/or the Centers for Disease Control and Prevention (CDC; Chan et al., 2010).

The long-term adaptation of pathogenic bacteria in eukaryotic cells (i.e., in bottleneck ecosystems), allowed them to become allopatric and specialists, and eventually to undergo reductive genome evolution (Merhej et al., 2009; Georgiades and Raoult, 2010). This dominant mode of evolution, in sequestrated intracellular parasites and symbionts from horizontal gene transfers, leads to a pseudogene-riddled genome, loss of non-essential genes and biosynthetic pathway components, as well as survival, by taking advantage of host cell metabolites (Andersson et al., 1998; Ogata et al., 2001; Audia and Winkler, 2006; Blanc et al., 2007a; Darby et al., 2007; Fournier et al., 2009; Sahni and Rydkina, 2009; Wolf and Koonin, 2013). Several intracellular pathogenic bacteria, including *Rickettsia, Mycobacterium*, and *Streptococcus* spp., have genomes smaller than less dangerous and cognate species, suggesting that enhanced virulence may be associated with reductive evolution (Demangel et al., 2009; Fournier et al., 2009; Merhej et al., 2014), rather than acquisition of virulence factors (Merhej et al., 2009; Georgiades and Raoult, 2010; Georgiades et al., 2011; Merhej et al., 2013).

Most molecular investigations on rickettsial-host interactions have identified several surface-exposed proteins (e.g., cell surface antigens, Scas), secretome and genes that may play fundamental roles in rickettsial infection pathogenicity and/or virulence (for reviews see Merhej and Raoult, 2011; Gillespie et al., 2015; Merhej et al., 2013; Sahni et al., 2013). However, recent genomic studies have narrowed the field of possible virulence factors of the *sca*5 (*omp*B) gene in *Rickettsia rickettsii* strains that differ in severity of disease (Clark et al., 2015). Moreover, a knockout of the *sca0* (cell surface antigens, *omp*A) gene in the virulent SFG *R. rickettsii* strain Sheila Smith concluded that this gene is not critical for virulence in the guinea pig model, but may play a role in survival or transmission from the tick vector (Ellison et al., 2008; Noriea et al., 2015). In another example, the *rick*A gene plays an important role in actin-based bacterial motility (Ogata et al., 2001; Gouin et al., 2004), a phenotype that has been associated with *Shigella* spp. and *Listeria monocytogenes* pathogenicity (Frischknecht and Way, 2001; Pollard and Borisy, 2003). However, the relationships between rickettsial pathogenicity and *rick*A remain questionable. Indeed, it is present in the avirulent and virulent *R. rickettsii* strains (Ellison et al., 2008), but also is pseudogenized, remnant, mutated or absent as *sca*0, 1 and/or 2 genes in the most pathogenic and non-motile species *R. prowazekii*, the less pathogenic and motile *R. typhi*, and/or in the non-pathogenic *Rickettsia peacockii* (Ogata et al., 2001; Balraj et al., 2008; Felsheim et al., 2009; Sears et al., 2012). Thus, understanding the mechanisms governing rickettsial pathogenicity and virulence outcomes in human hosts needs to be elucidated, using integrative and modern gel-free omics approaches before for example gel-based or isogenic methods. As an example, in *Mycobacterium tuberculosis*, the LC-MS/MS proteomic analysis identified high numbers of proteins (from 691 to 983), of which several were up-regulated, down-regulated or unique during the dormancy and reactivation of a virulent strain (Gopinath et al., 2015).

This study focused on four SFG *Rickettsia* species exhibiting differences in ecologic and biologic features, in which the genetic basis remains sparse. *Rickettsia slovaca* and *R. raoultii*, which are mainly associated with *Dermacentor* ticks, cause scalp eschar and neck lymphadenopathy (SENLAT) in humans (Raoult and Roux, 1997; Parola et al., 2009). *Rickettsia conorii* and *Rickettsia massiliae*, which are most often associated with *Rhipicephalus* ticks (Parola et al., 2013), cause Mediterranean spotted fever (MSF) in humans (Milhano et al., 2014; Bechelli et al., 2015; Portillo et al., 2015). However, *R. slovaca* and *R. conorii* share the common characteristics of being both less prevalent in their respective tick vectors and more pathogenic for humans than their counterparts. In *Dermacentor* ticks, *R. slovaca*, and *R. raoultii* exhibit prevalence of 0–4 and 10–82%, respectively (Parola et al., 2009; Milhano et al., 2010; Jiang et al., 2012; Speck et al., 2012; Spitalska et al., 2012; Wen et al., 2014) but in humans, they are detected in 57 and 8% of SENLAT cases, respectively (Parola et al., 2009; Foissac et al., 2013). Similarly, *R. conorii* and *R. massiliae* exhibit a prevalence of 0–0.7 and 8–17% in *Rhipicephalus* ticks (Fernandez-Soto et al., 2006a,b; Marquez et al., 2008), whereas the former is highly virulent and causes severe MSF with a mortality rate up to 30%, and the latter only causes a mild MSF disease (Cascio et al., 2013; Bechelli et al., 2015). Thus, to identify potential genes and protein profiles, as well as to understand the driving forces that could, comprehensively, relate to the differences in virulence and pathogenicity in humans of these four SFG *Rickettsia* species, we compared their genomic sequences and proteomic profiles and examined their evolutionary relationships.

## Materials and methods

### Genomic analysis

Herein, we studied four rickettsiae, including the *Dermacentor*-transmitted species *R. slovaca* strain 13-B^T^ (CSUR R154, Fournier et al., 2012) and *R. raoultii* strain Khabarovsk^T^ (CSUR R3, ATCC VR-1596, El Karkouri et al., 2016) that cause SENLAT, and the *Rhipicephalus*-associated *R. conorii* strain Malish 7^T^ (CSUR R41, ATCC VR-613, Ogata et al., 2001) and the *R. massiliae* strain MTU5 (CSUR R132, Blanc et al., 2007b) causing MSF. For each disease, the former species caused a more severe infection. The four species were obtained from the French “Collection de Souches de l'Unité des Rickettsies” (CSUR).

Genomic sequences of the four species were downloaded from the NCBI FTP server (ftp://ftp.ncbi.nih.gov/Genome/). To avoid potential biases across the originally published data, including unpredicted Open Reading Frames from pseudogenes in the GenBank database that were generated by different gene identification and annotation systems, all genomes were subjected to a standard re-annotation, including CDSs (coding sequences) prediction with the AMIGene software (Bocs et al., 2003). The automatic assignment of protein functions was performed against the RickBase (Blanc et al., 2007a) and non-redundant NR databases using PipRick (an in-house annotation pipeline written in Perl language) and BLASTp algorithm (Altschul et al., 1997). The annotations were then curated and genes that were either complete or altered (split or fragment) were distinguished (Blanc et al., 2007a). Functional classification of gene families (COG ID and Letters) was searched using COGsoft software against the Clusters of Orthologs Groups (COG) database (Kristensen et al., 2010). The pan-genome between the four species was constructed by subjecting predicted proteomes to a reciprocal best BLAST hit (BBH) algorithm with all-against-all search (coverage of the query length ≥60% and *E-value* < 10^−10^) using COGsoft software. Each cluster of orthologous groups of rickettsial genes from chromosomes and plasmids was named cRIGs and pRIGs, respectively. The Venn diagrams of pan-genome and pan-proteome were constructed using the Jvenn Javascript library (Bardou et al., 2014). To identify putative virulence factors, a BLASTp search was performed against the virulence factor database, VFDB (Chen et al., 2016). Multiple sequence alignments of the core genes were carried out using MAFFT software (Katoh et al., 2005). Phylogenetic analysis was performed with the maximum likelihood (ML) method under the JTT amino acid substitution matrix, the Nearest-Neighbor-Interchange (NNI), the gamma (Γ) distribution of parameter α to account for substitution rate heterogeneity among sites and complete deletion and the rectangular tree using MEGA software (Tamura et al., 2013). Moreover, a neighbor-joining (NJ) tree was constructed from a gene content distance matrix calculated according to the pan-genome data and Jaccard's dissimilarity coefficient (El Karkouri et al., 2006). The robustness of the nodes in both ML and NJ trees was estimated through Bootstrap (BP) analyses of 100 and 500 replicates with MEGA software and the PHYLIP package (Website: http://evolution.genetics.washington.edu/phylip.html), respectively. To assess the genomic differences in predicted proteins of the core genes between the four species, the percentage of the amino acid identities and the numbers of non-synonymous mutations and insertions/deletions (InDels) were computed using the Smith-Waterman BLASTp search algorithm. For this four-way analysis, the *E-value* cutoff < 10^−10^ was used and the false positive matches were removed.

### *Rickettsia* culture and purification

A confluent monolayer of the *Xenopus laevis* cell line (XTC-2 cells) in Leibovitz's L-15 medium supplemented with tryptose phosphate buffer (5%) and fetal bovine serum (4%; Life Technologies) was inoculated with a *Rickettsia* species, as described by Saisongkorh et al. (2012). For each studied *Rickettsia* species, about 25 × 10^4^–5 × 10^5^ bacteria were inoculated in *X. laevis* cells (one 150 cm^2^ flask each, containing 25 ml of fresh medium) and incubated at 28°C for 5 days. Then, each species was subcultured in 20 flasks prior to being collected, pooled and purified individually on a discontinuous renografin gradient, as previously reported (Eremeeva et al., 1994). Purified rickettsiae counted 1.6 × 10^7^ bacteria for *R. slovaca*, 5 × 10^10^ bacteria for *R. conorii*, 2.4 × 10^10^ bacteria for *R. raoultii* and 3.4 × 10^9^ bacteria for *R. massiliae*. They were then washed in PBS at 10,000 × g at 4°C for 10 min, and then stored at −80°C for further analysis. All infection and purification steps were monitored by Gimenez staining (Gimenez et al., 1964). Bacterial quantities of both steps were assessed using quantitative real-time PCR (qPCR) with the 1,029 system based on the RC0338 hypothetical protein gene of all SFG rickettsiae (Socolovschi et al., 2012).

### Preparation of proteins for nano-LC/MS/MS

Aliquots of purified bacteria of each species were pelleted, PBS discarded and then resuspended in 200 μl of lysis buffer (7 M urea, 2 M thiourea, 4% w/v CHAPS, 30 mM Tris-HCl, pH 8.0) and lysed by sonication (Vellaiswamy et al., 2011). Soluble proteins were then dialyzed twice using Slide-A-Lyzer Dialysis Cassettes 2K MWCO (Pierce Biotechnology, Rockford, USA) against 1 L of 50 mM ammonium bicarbonate pH 7.4, 1 M urea (7 h and overnight). The total soluble proteins of dialyzed fractions were quantified by Bradford assay (Biorad, Marnes-la-Coquette, France). Disulphide bonds were reduced by treating 50 μg of soluble proteins of each sample with 10 mM DL-dithiothreitol (Euromedex, Souffelweyersheim, France) in 50 mM ammonium bicarbonate (Sigma, Saint-Quentin Fallavier, France) buffer at room temperature for 1 h. The proteins were subsequently alkylated with 20 mM iodoacetamide (Sigma, Saint-Quentin Fallavier, France) in the same buffer at room temperature in the dark. The alkylated proteins were then digested with 2 μg of sequencing-grade trypsin (Promega, Charbonnières, France) overnight at 37°C. The digested peptide solutions were then desalted using Pierce Detergent Removal Spin Columns (Thermo Fisher Scientific, Illkirch, France) and stored at −20°C until the LC/MS/MS analysis.

### Liquid chromatography and mass spectrometry

The peptide samples were analyzed using a nano ACQUITY 2D-UPLC system coupled to a Quadrupole Time-of-Flight (Q/TOF) traveling wave ion mobility hybrid mass spectrometer (SYNAPT-G2SI, Waters, Guyancourt, France). Both systems were operated and controlled by MassLynx4.1 software (Waters, Guyancourt, France). All solvents used were ULC-MS grade (Biosolve, Dieuze, France). Each peptide sample was run in three analytical replicates. The digested peptide solutions (equivalent to about 1.4 μg of proteins/μl) were 6-time diluted and spiked with 10 fmol/μl of digested yeast alcohol dehydrogenase (ADH; MassPREP, Waters, Guyancourt, France). Briefly, 3 μl of each peptide sample, equivalent to about 700 ng of proteins was fractionated using a nano 2D chromatography setup and then monitored using a high definition HD-MS^E^ method (Waters, Guyancourt, France), as previously described (Reteno et al., 2015). Briefly, peptides were collected on a first reverse phase column at high pH 10, and then, seven eluting peptide fractions were successively trapped after an online dilution. Each fraction was separated at low pH 2.5 on a second reverse phase column. The mass spectrometry proteomics data have been deposited in the ProteomeXchange Consortium (Vizcaino et al., 2014) via the PRIDE partner repository with the following dataset identifiers: PXD003193, PXD003194, PXD003195, and PXD003197.

### Data processing and analysis

Raw MS^*E*^ data from each biological sample were processed using the ProteinLynx Global SERVER v3.0.1 (PLGS, Waters, Guyancourt, France) for protein identification and protein quantification. Noise reduction thresholds for low energy scan ion, high energy scan ion and peptide intensity were set at 1,000, 100, and 800 counts, respectively. A mass correction was applied to all spectra using the Leucine Enkephalin lock mass calibrant at 785.8426 m/z. For each sample, a single mass spectrum file was generated by merging the mass spectra from the seven fractions. To perform the protein sequence database search, we constructed four distinct databases, each containing annotated protein sequences of one *Rickettsia* species. For each database, we also included protein sequences of the *X. laevis* species downloaded from the NCBI database, the common contaminants and the yeast ADH sequence (accession number: P00330|ADH1) from the universal protein Knowledgebase, UniProt (UniProt, 2017). The default parameters used for global peptide and protein identifications were set as follows (see Gopinath et al., 2015): at least one fragment ion match per peptide, at least three fragment ion matches per protein, at least one peptide matches for protein identification, mass tolerances was set to automatic with a window of 10 ppm for precursor ions and a window of 20 ppm for fragment ions, at least one positive charge, oxidation of methionine (M) as variable modification and carbamidomethylation (C) of cysteine as the fixed modification, and the trypsin was selected as the enzyme with up to one miss-cleavage. The initial protein false discovery rate (FDR) of the identification algorithm was set at 4% with a randomized database, leading to a peptide FDR that was typically smaller than 1% (Brioschi et al., 2013; Gopinath et al., 2015). Protein quantities were evaluated in the injected solution using the combined intensity of the three most abundant peptides per protein compared to the quantitatively added yeast ADH digest (Hi3 absolute quantification, PLGS, Waters, Guyancourt, France; Silva et al., 2006).

Only proteins identified by at least two matched peptides were considered for proteomic analysis (Treitz et al., 2015). As the three replicates of each sample showed high reproducibility with high significant linear correlations (Spearman's and Pearson's correlations, *P* < 0.001), we grouped each three replicates and calculated the average abundances (fmol or fmol μg^−1^) of each protein. The fmol averages of each sample were also normalized by the median using the Perseus Software (v1.5.1.6; www.maxquant.org, Treitz et al., 2015). Moreover, using each protein's molecular weight from the database, the fmol quantity of each protein was converted to nanograms for the triplicates, and by summing the average ng of all proteins we obtained the total average ng in each sample (Saka et al., 2011). To calculate the species protein abundance for each sample, the fmol average for each protein was divided by the average ng sum for only the sample of that species, and scaled by 1,000 to yield fmol μg^−1^ (Saka et al., 2011). The ratios describing the condition virulent/milder species of two comparative models (*R. slovaca*/*R. raoultii* and *R. conorii*/*R. massiliae*) were then log_2_ transformed. Orthologous proteins with stringently defined fold change (FC ≥ 2) were considered to represent up-regulation (log_2_ ratio ≥ 1) or down-regulation (log_2_ ratio ≤ −1), whereas orthologous proteins with a FC < 2 were considered as equally regulated (Son et al., 2015). Proteins with FC = 2 included a minimal and a well-represented abundance value of 0.5 fmol. Thus, orthologous proteins with stringent abundance values (≥0.5 fmol) in one species, but with zero abundance values in the second species, were also comprehensively annotated as up-regulated or down-regulated. In contrast, proteins with these abundance values (≥0.5 fmol) in only one species (i.e., the gene is absent in the second species) were annotated as specific or unique (Gopinath et al., 2015). Other proteins that did not match these criteria were annotated as un-classified.

### Statistical analysis

Statistical analyses were processed using the R Commander software (http://r-forge.r-project.org). These included Pearson's and Spearman's correlation coefficients (R and Rho, resp.), Chi-square test (χ^2^) and Fisher's exact test.

## Results

### Genomics of SFG *Rickettsia* spp.

**Figure 1:**
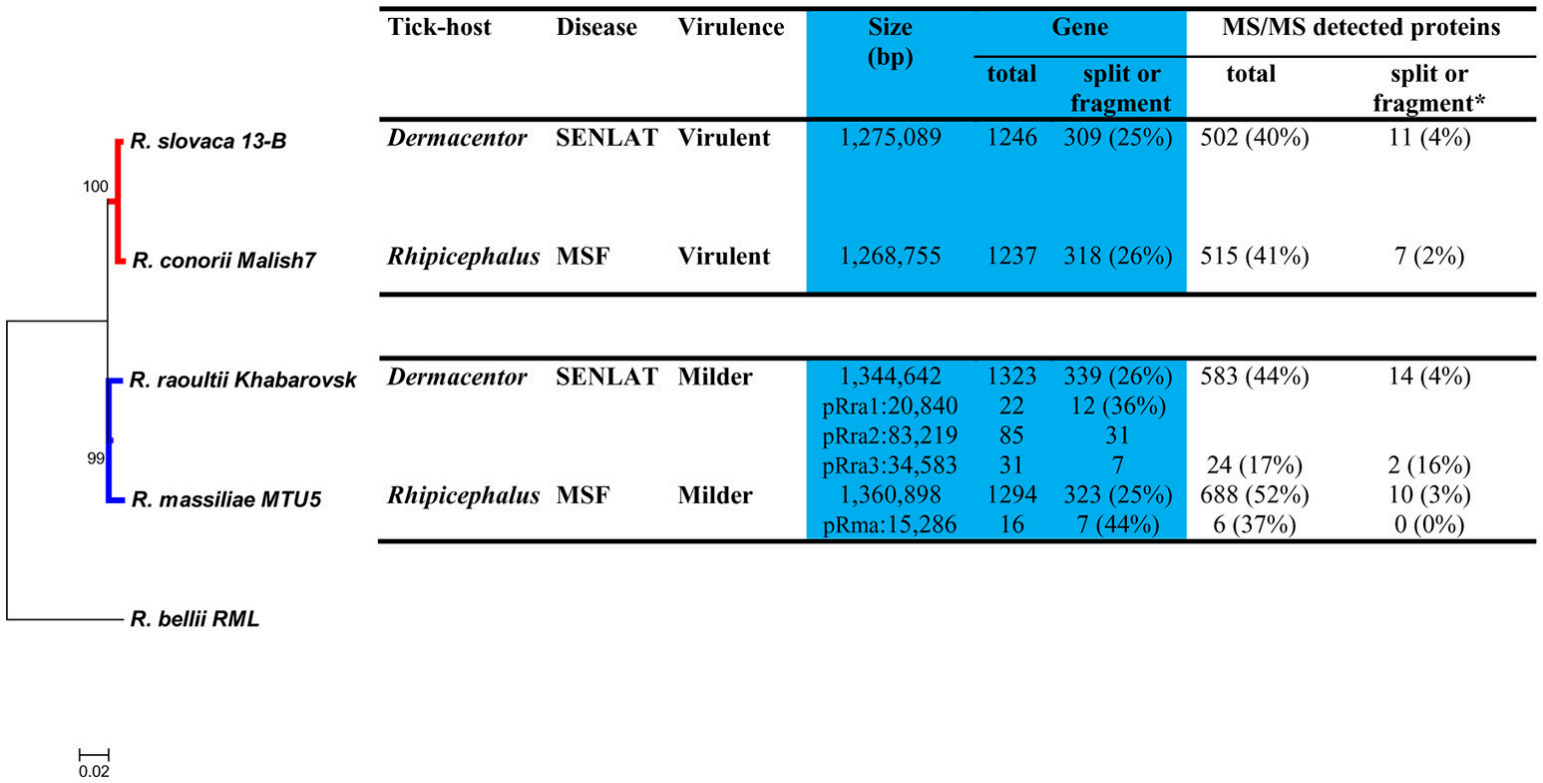
Phylogenomic tree and, biologic, pathogenic, genomic, and proteomic features, of four SFG *Rickettsia* species. Bootstrap supports higher than 90% are shown at the nodes. ^*^Means that the total of the detected MS/MS proteins corresponded to genes which are either split or fragment.

The general features of the four *Rickettsia* genomes are summarized in Figure 1. The chromosomes of the virulent *R. slovaca* and *R. conorii* agents were from 70 to 92 kbp smaller than those of the milder *R. raoultii* and *R. massiliae* agents. The former species are plasmidless, whereas the latter harbored one to three plasmid(s) (15–83 kbp). Moreover, about 74–75 and 56–64% of genes in the chromosomes and plasmids were found complete, respectively, whereas the remaining genes were either split or fragments.

**Figure 2:**
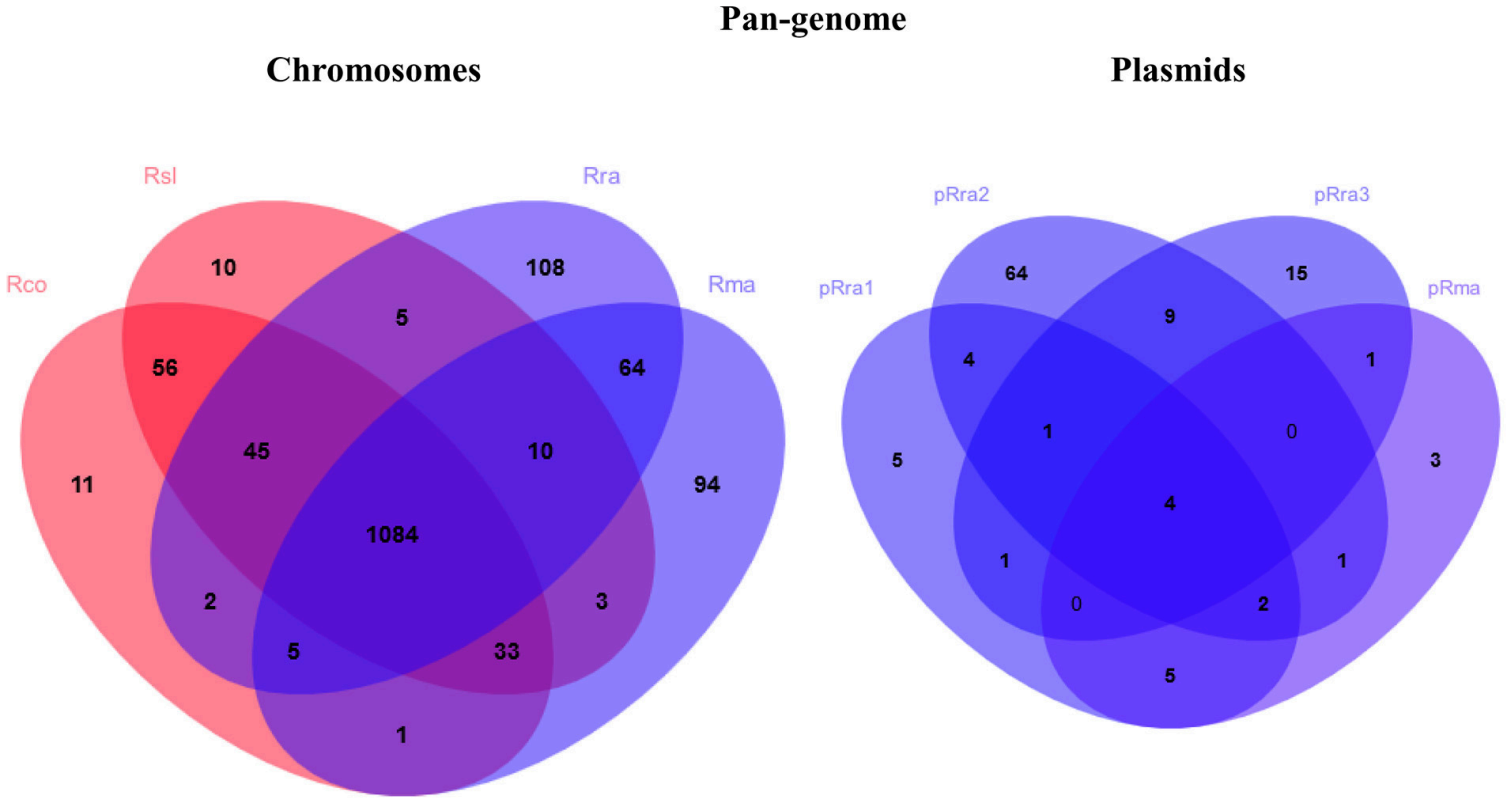
Venn diagrams summarizing pan-genome of the virulent *R. slovaca* Rsl and the milder *R. raoultii* Rra causing SENLAT, and the virulent *R. conorii* Rco and the milder *R. massiliae* Rma causing MSF.

The pan-genome analysis from the four *Rickettsia* spp. identified 1,531 and 115 clusters of rickettsial orthologous genes from the chromosomes (cRIGs) and plasmids (pRIGs), respectively (Figure 2). The four species displayed a core-chromosome of 1,084 (71% of 1,531) cRIGs. The pairwise Smith-Waterman similarity search in the 1,084 core genes revealed that the two virulent agents exhibited more conservation in their core genes with a high average aa identity and significant low numbers of non-synonymous (NS) mutations and InDels (Rsl vs. Rco: 98%, 3,335 and 933, resp.) when compared with the two milder species (Rra vs. Rma, 96%, 7,888 and 1,495, *P* ≤ 0.001, χ^2^ test, resp.). In the four-way comparisons between one virulent and one milder agent (e.g., Rsl vs. Rra), the conservations in the core genes (average aa identity: 95%) were found to be higher and slightly lower than those obtained between the two virulent agents (98%) and between the two milder agents (96%), respectively. Moreover, the total numbers of NS mutations and InDels observed between one virulent and one milder agent (Rsl vs. Rra: 8,756 and 1,442, resp.; Rsl vs. Rma: 9,126 and 1,659, resp.; Rco vs. Rra: 9,470 and 1,471, resp.; Rco vs. Rma: 9,808 and 1,523) were significantly higher than those found between the two virulent agents (Rsl vs. Rco: 3,335 and 933, resp.) (*P* ≤ 0.001, χ^2^ tests) and between the two milder agents (Rra vs. Rma, 7,888 and 1,495, resp.) (*P* ≤ 0.001, χ^2^ tests).

The phylogenomic tree performed from 330 concatenated core genes revealed that the four pathogenic species diverged from a common ancestor into two major clades that distinguish the virulent species (BP = 100%) from the milder species (BP = 97%; Figure 1). This divergence appears to be more ancient than those which occurred from the common ancestor of the two virulent species and that of the two milder agents. However, this tree did not distinguish the *Dermacentor*-associated rickettsiae causing SENLAT (*R. slovaca* and *R. raoultii*) from the *Rhipicephalus*-associated rickettsiae causing MSF (*R. conorii* and *R. massiliae*). The neighbor-joining tree displayed high gene content dissimilarities (0.20) between the two virulent and the two milder agents (Figure S1). Moreover, the former and the latter agents exhibited very low (0.01) and high (0.20) dissimilarity values, respectively.

**Figure 3:**
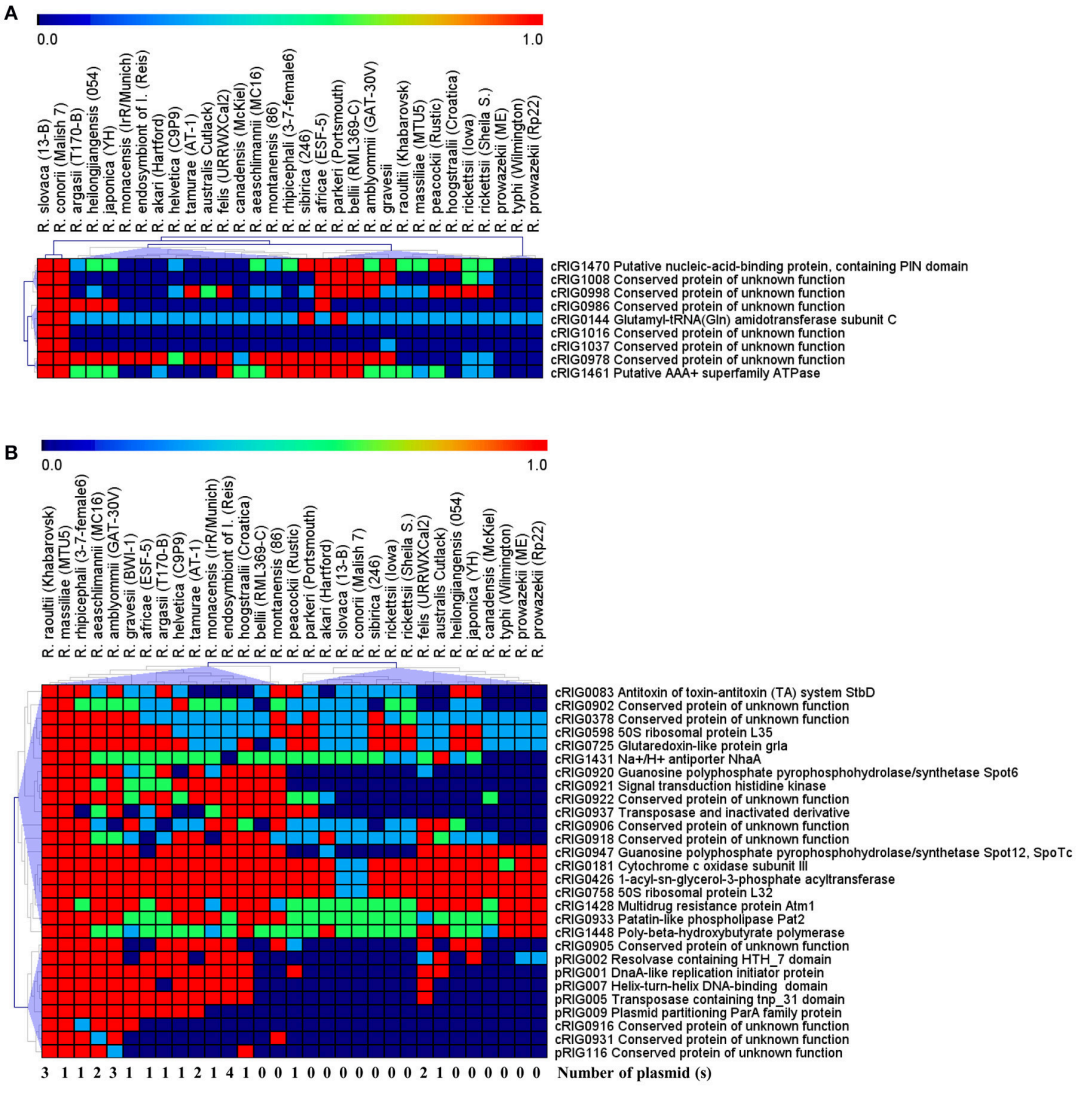
Comparative genomics of 9 complete genes (≥60 aa in sizes) distinguishing the virulent *R. slovaca* and *R. conorii* from the milder *R. raoultii* and *R. massiliae* **(A)** and 28 complete genes (≥60 aa in sizes) distinguishing the milder *R. raoultii* and *R. massiliae* from the virulent *R. slovaca* and *R. conorii* **(B)**, with 25 *Rickettsia* species. Red, green, light blue, and dark blue colors mean that genes can be either complete, split, fragment and absent/remnant, respectively.

Examination of gene degradation and loss revealed that the two virulent agents have altered or lost more genes (92 of 1,161 core genes) than the two milder agents (60 of 1,140 core genes; *P* = 0.003, χ^2^ test). This also means that the virulent species shared 60 core genes (with 37–881 aa in sizes) that were deleted or altered in both the milder species (Figure 2, Figure S2A). Of these, comparative genomics followed by hierarchical clustering of 9 complete genes (with ≥60 aa in size) between 29 *Rickettsia* spp. revealed that they were also either complete, altered or lost in less pathogenic species (e.g., *R. peacockii*, and *Rickettsia africae*) and avirulent strains (*R. rickettsii* Iowa and *R. prowazekii* ME) as well as in more pathogenic species (e.g., *R. rickettsii* Sheila, *R. australis, R. prowazekii* and *R. typhi*; Figure 3A). None of these genes (except one ankyrin repeat-containing protein which is split, cRIG1027) has found any BLAST match with any putative virulence factor previously described in the genus *Rickettsia*, and none of them in the virulence factor database of pathogenic bacteria (VFDB). Inversely, the milder species harbored 92 core genes (with 38–2,500 aa in size) that were lost or altered in the virulent species (Figure 2, Figure S2B). Of these, comparative genomics followed by hierarchical clustering of 28 complete genes (with ≥60 aa in size) between 29 *Rickettsia* spp. showed two major clusters (Figure 3B). The first cluster included 48% of rickettsial species displaying several genes in a gradual degradation process or lost. Among these species, we found the two virulent species (*R. conorii* and *R. slovaca*), several more pathogenic species (e.g., *R. rickettsii* Sheila, *R. prowazekii* Rp22, and *R. typhi*), and only three nonpathogenic strains *R. peacockii* Rustic and the two mutants *R. rickettsii* Iowa and *R. prowazekii* ME. In contrast, the second cluster contained 52% of rickettsial species exhibiting several conserved genes. Among these species, we found the two milder species (*R. raoultii* and *R. massiliae*) and several other less or non-pathogenic species (e.g., *R. africae, R. rhipicephalus, R. montanensis*). Moreover, the first cluster also gathered 11 plasmidless species, and only three species with one plasmid, including *R. peacockii, R. australis*, and *Rickettsia felis*, whereas the second cluster included 13 species harboring one to four plasmids, but only two plasmidless species, including *R. bellii* and *R. montanensis* (*P* = 0.01, Fisher's exact test). Comparative COG categories showed that the two virulent species mainly lacked mobilome genes relative to the two milder species (5 vs. 29–41 genes per sp.; Figure S3). Moreover, only the latter species exhibited additional genes in several COG categories from plasmids, including for example 2–11 genes from the mobilome. In all, the numbers of genes in the mobilome found in the virulent *R. slovaca* or *R. conorii* differed significantly from those of the milder *R. raoultii* or *R. massiliae*, respectively (5 of 1,246 vs. 40 of 1,323, *P* ≤ 0.001, or 5 of 1,237 vs. 43 of 1,294, *P* ≤ 0.001, resp., χ^2^ tests).

### Proteomics of SFG *Rickettsia* spp.

Proteome analysis of the four SFG species identified 502–688 and 6–24 chromosomes- and plasmids-encoding proteins after infecting *X. laevis* cells, respectively. These proteins covered 40–52% (of 1,246–1,294) and 17–37% (of 16–138) of the four predicted proteomes, respectively (Figure 1). Although these numbers of identified proteins may appear low, they are similar to those observed strictly intracellular bacteria such as *Chlamydia trachomatis* (Saka et al., 2011). Expressions from genes, either split or fragment, were rare, counting about 2–4% (7–14 of 302–334 genes split or fragment) from chromosomes and 0–16% (0–2 of 7–12 genes split or fragment) from plasmids.

**Figure 4:**
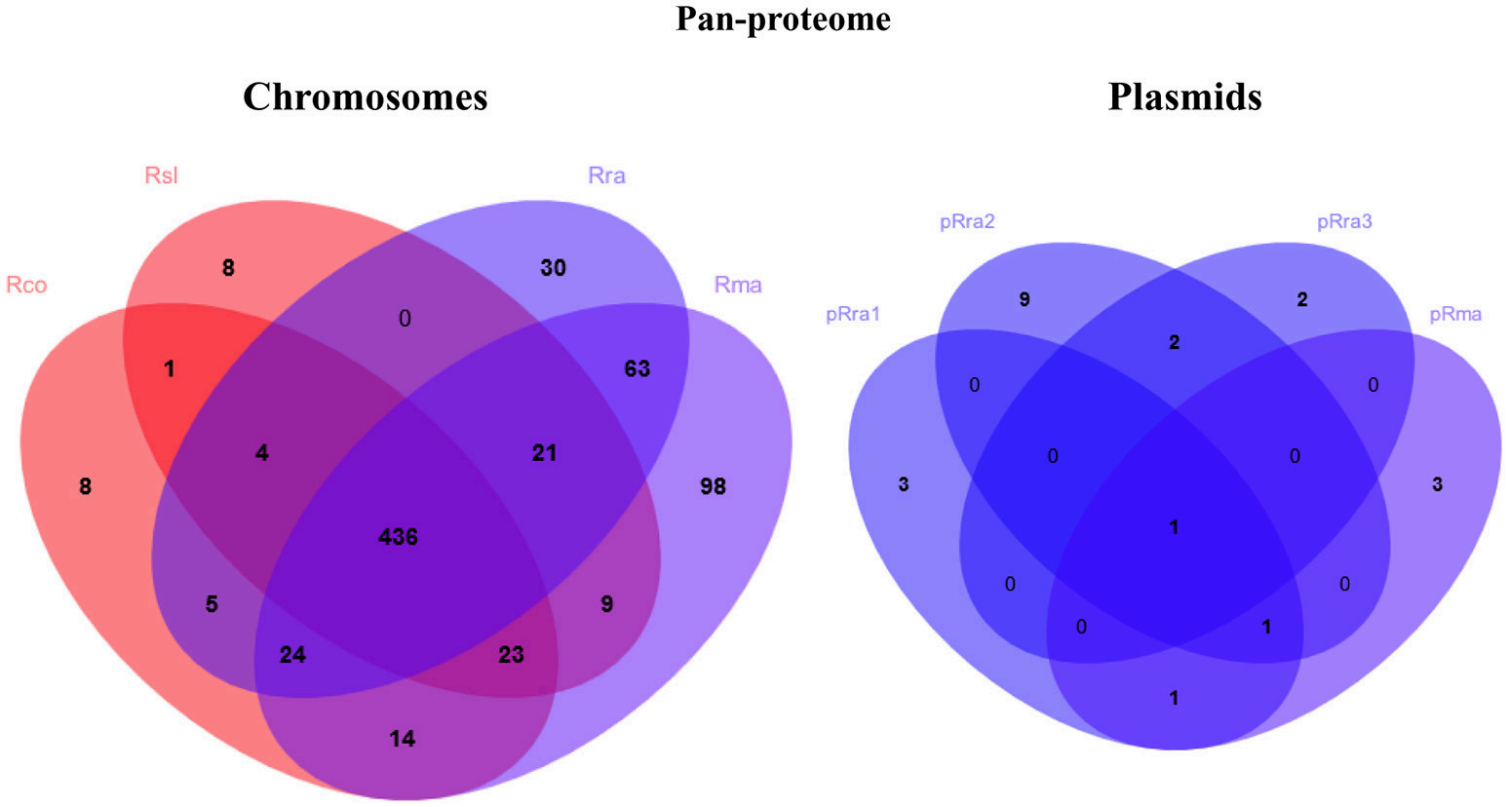
Venn diagrams summarizing pan-proteome of the virulent *R. slovaca* Rsl and the milder *R. raoultii* Rra causing SENLAT, and the virulent *R. conorii* Rco and the milder *R. massiliae* Rma causing MSF.

The pan-proteome of the four *Rickettsia* spp. clustered into 744 cRIGs and 22 pRIGs (Figure 4). Of these, only 210 (28% of 744 cRIGs) and only 2 (10% of 22 pRIGs) of identified proteins were previously detected in diverse studies using gel-based proteomics, or rarely RT-PCRs (Table S1). However, 534 (72% of 744 cRIGs) and 20 (90% of 22 pRIGs) of expressed proteins are newly identified by the current gel-free proteomics (Table S1). Moreover, 96% (717 of 744 cRIGs) of the pan-proteome represented 66% of genes from the core chromosome (717 of 1,084 cRIGs), while only 4% (27 of 744 cRIGs) were found in up to three species from chromosomes. Only 19% (22 of 115 pRIGs) of genes in plasmids exhibited expressions.

**Figure 5:**
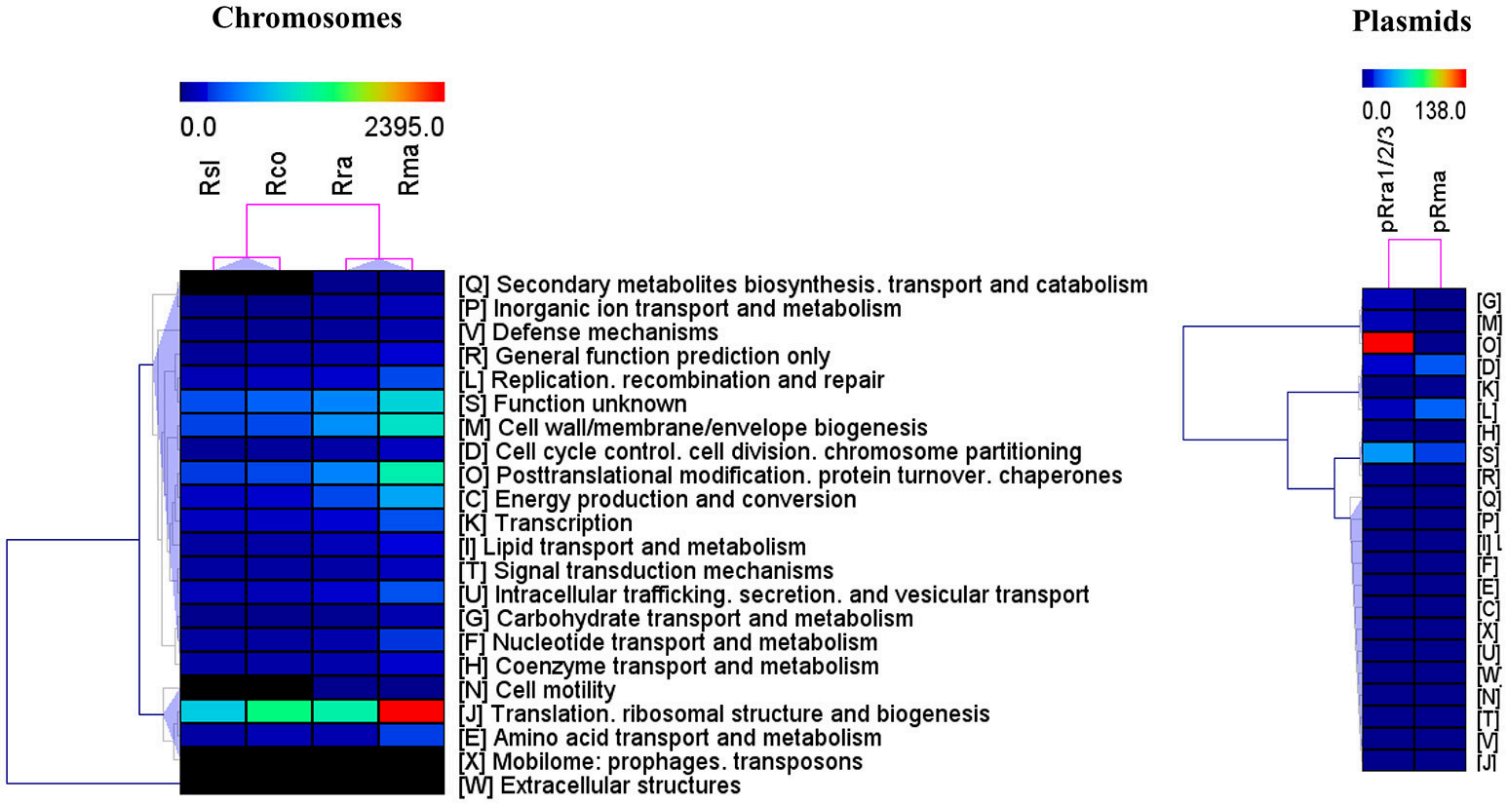
Hierarchical clustering of total protein quantities (fmol μg^−1^) in the two virulent species *R. slovaca* Rsl and *R. conorii* Rco, and, the two milder species *R. raoultii* Rra and *R. massiliae* Rma, as classified by COG functional categories.

The pan-proteome of the four pathogens displayed a number of identified core proteins, significantly lower in the virulent than in the milder species (1 of 437 vs. 66 of 502 c/pRIGs; *P* < 0.001, χ^2^ test; Figure 4, see protein list in Table S2). Moreover, the total abundance of these proteins were lower (1.3–2.7 fmol μg^−1^) in the virulent than in the milder species (230.5–516.4 fmol μg^−1^; Table S2). Moreover, 90% of these proteins were coded by core genes. All protein abundances of the four species are given in Table S3. Hierarchical clustering analysis of total protein abundances, classified by COG categories, also clearly distinguished the virulent from the milder species (Figure 5). This distinction was found mainly in proteins involved in post-translational modification, protein turnover and chaperones (282–327 vs. 694–1156 fmol μg^−1^, resp.), cell wall/membrane/envelope biogenesis (317–332 vs. 615–1040 fmol μg^−1^, resp.), and energy production and conservation (172–206 vs. 331–712 fmol μg^−1^, resp.).

**Figure 6:**
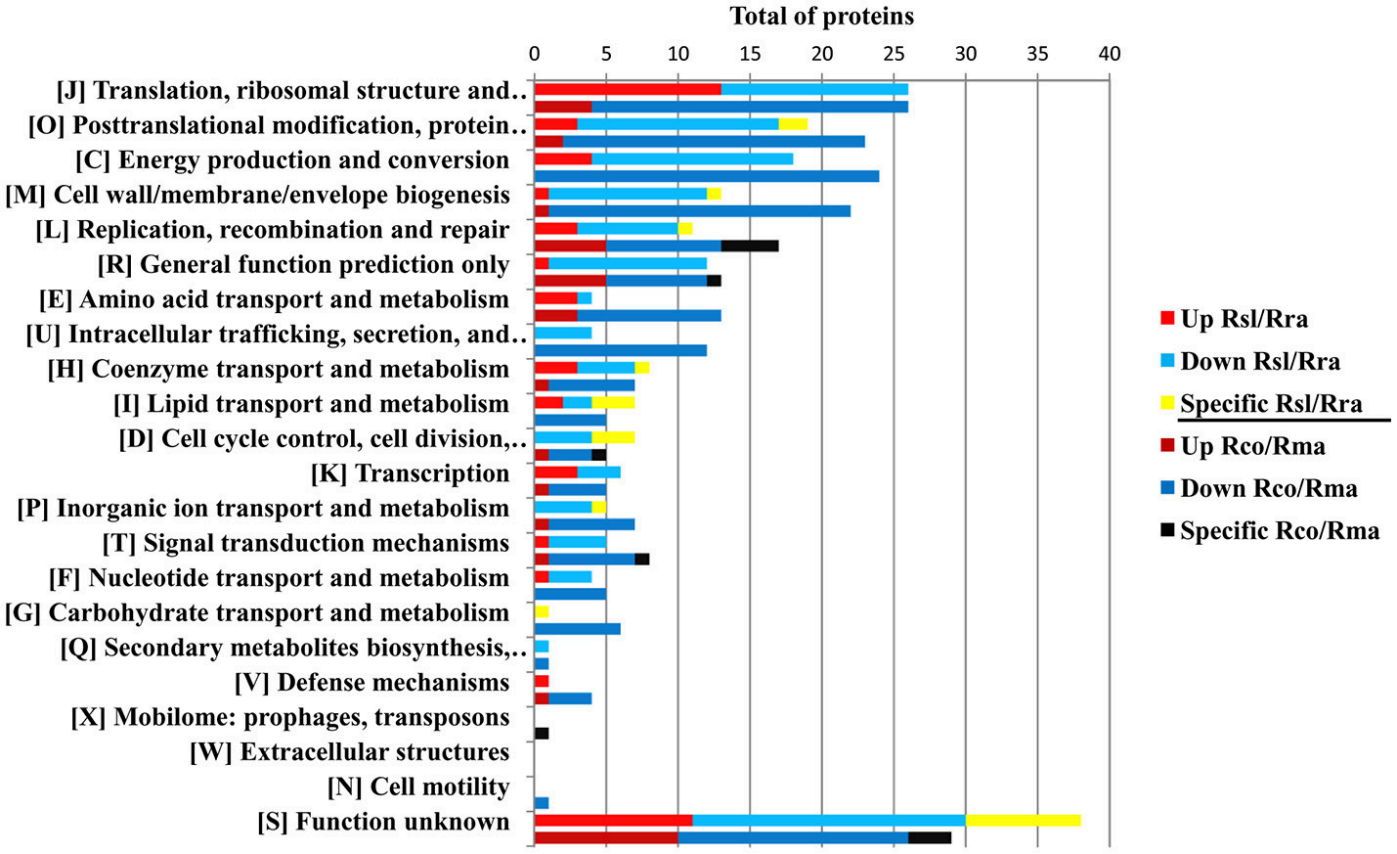
Protein profiles obtained between the SENLAT agents (the virulent *R. slovaca* Rsl/the milder *R. raoultii* Rra), and between the MSF agents (the virulent *R. conorii* Rco/the milder *R. massiliae* Rma), as classified by COG functional categories. Up, Down and Specific mean up-regulated down-regulated and specific proteins.

**Figure 7:**
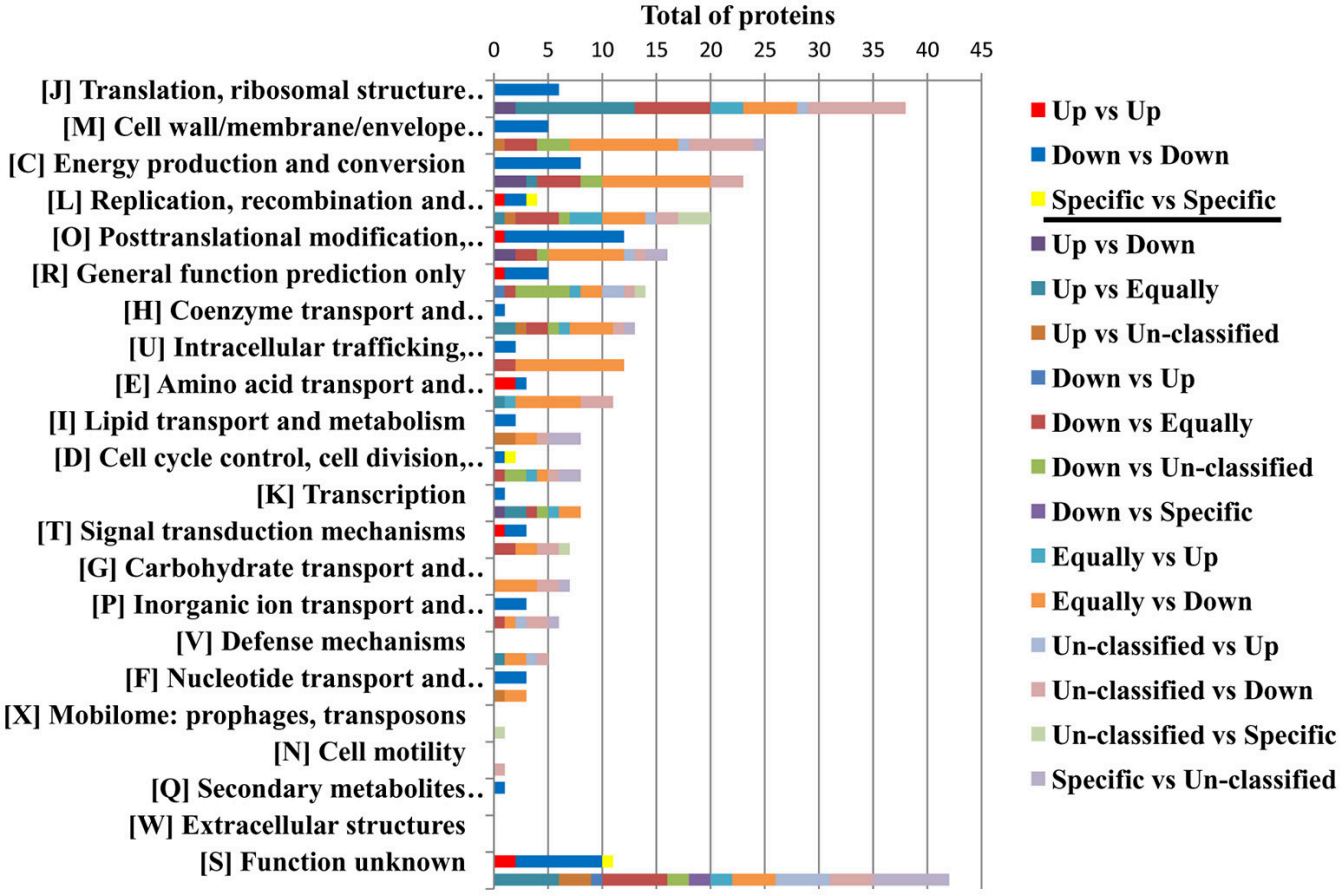
Similar and distinct protein patterns obtained between the SENLAT agents (the virulent *R. slovaca* Rsl/the milder *R. raoultii* Rra) and the MSF agents (the virulent *R. conorii* Rco/the milder *R. massiliae* Rma), as classified by COG functional categories. Up, Down, Equally, Specific and Un-classified mean up-regulated, down-regulated, equally regulated, specific and un-classified proteins, respectively.

The protein profile of the virulent compared with the milder SENLAT agents identified 169 core proteins (26% of 643 c/pRIGs) exhibiting abundance changes, in which the virulent *R. slovaca* up-regulated 50 and down-regulated 119 core proteins (Figure S4). Moreover, only the milder *R. raoultii* displayed 21 (3.2% of 643 c/pRIGs) specific proteins, including 17 from plasmids. Similarly, the protein profile of the virulent compared with the milder MSF agents resulted in 223 core proteins (31% of 712 c/pRIGs) displaying abundance changes, in which the virulent *R. conorii* up-regulated 36 and down-regulated 187 core proteins (Figure S4). Moreover, the virulent and the milder agents each exhibited one and 10 specific proteins, including 3 from plasmids (1.5% of 712 c/pRIGs), respectively. Overall, both the virulent compared with both the milder agents exhibited less up- than down-regulated proteins and rarely specific proteins (50–36 vs. 119–187 vs. 21–10, *P* < 0.001, χ^2^ test). Examination of the protein profiles by COG categories revealed that both virulent agents displayed large numbers of proteins with changes in abundances and/or specificity in translation, ribosomal structure and biogenesis (26 and 26 proteins), post-translational modification, protein turnover, chaperones (19 and 23 proteins), energy production and conversion (18 and 24) and general function prediction (12 and 13 proteins; Figure 6). However, the virulent SENLAT agent exhibited a number of proteins, with changes in abundances or specificity, slightly lower than those of the virulent MSF agent, mainly in cell wall/membrane/envelope biogenesis (13 vs. 22 proteins) and replication, recombination and repair (11 vs. 17 proteins), intracellular trafficking, secretion, and vesicular transport (4 vs. 12 proteins) and amino acid transport and metabolism (4 vs. 13 proteins; *P* < 0.6, χ^2^ test). Comparative analysis of protein profiles between the SENLAT and MSF agents identified 340 c/pRIGs, distinguishing two main patterns. First, the two virulent agents shared 72 proteins (21% of 340 c/pRIGs) exhibiting similar patterns, in which we found 8 up/up-regulated and 61 down/down-regulated proteins, as well as three plasmid-specific proteins of the two milder species (Figure S5). These patterns were found more in proteins associated with post-translational modification, protein turnover, chaperones (12 proteins) and energy production and conversion (8 proteins) than those related to translation, ribosomal structure and biogenesis (6 proteins), cell wall/membrane/envelope biogenesis (5 proteins) and general function prediction only (5 proteins; Figure 7, Table 1, Table S4). In the remaining categories, the similar patterns displayed low numbers of or no proteins. Second, and in contrast, the two virulent agents exhibited 268 proteins (79% of 340 c/pRIGs) displaying distinct protein patterns (Figure S5). As an example, while the virulent SENLAT agent up-regulated 42 proteins, the virulent MSF agent did not up-regulate them, but carried out down-regulations for 8, equal regulations for 25 and un-classified proteins for 9 core proteins. In all, while the virulent SENLAT agent exhibited 42 up-regulated, 58 down-regulated, 91 equally regulated, and 59 un-classified proteins, as well as 18 proteins specifically expressed by the milder agent, the virulent MSF agent displayed 28 up-regulated, 126 down-regulated, 61 equally regulated, and 45 un-classified proteins, as well as 8 specific proteins, including one by the virulent agent and 7 by the milder agent (*P* < 0.001, χ^2^ test). These distinct patterns were found more in proteins related to translation, ribosomal structure and biogenesis (38 proteins), cell wall/membrane/envelope biogenesis (25 proteins), energy production and conversion (23 proteins), replication, recombination and repair (20 proteins), post-translational modification, protein turnover, chaperones (16 proteins) and general function prediction only (14 proteins), than in those associated with coenzyme transport and metabolism (13 proteins), intracellular trafficking, secretion, and vesicular transport (12 proteins), amino acid transport and metabolism (11 proteins; Figure 7, Table 1, Table S4). In the remaining categories, the distinct patterns exhibited low numbers of proteins.

**Table 1:** Examples of protein patterns obtained between the SENLAT agents (the virulent *R. slovaca* Rsl/the milder *R. raoultii* Rra) and between the MSF agents (the virulent *R. conorii* Rco/the milder *R. massiliae* Rma).

**Diseases**			**SENLAT**	**MSF**
**Cluster ID**	**Protein function**	**Gene**	**Rsl/Rra**	**Rco/Rma**
**SIMILAR PATTERNS**				
cRIG0680	RmuC family protein	–	+3.7	+3.3
cRIG0232	**Regulatory components of sensory transduction system**	–	+3.0	+3.0
cRIG0681	ATP-dependent clp protease ATP-binding subunit ClpX	*clpX*	+2.8	+3.3
cRIG0146	**Ribosome recycling factor**	***rrf***	−6.2	−8.8
cRIG0661	**Cold shock-like protein**	***cspA***	−7.2	−5.7
cRIG0808	***Rickettsia*** **adhesin Adr2**	***Adr2***	−2.8	−2.5
cRIG0218	**Type I secretion outer membrane protein TolC**	***tolC***	−3.0	−2.5
cRIG0427	Host attachment protein AtsE-like	–	−6.5	−16.8
cRIG0002	**Thioredoxin**	***trxA***	−2.9	−21.2
cRIG0618	**10 kD chaperonin**	***GroES***	−2.6	−4.2
cRIG0421	Aminomethyltransferase folate-binding domain	*ygfZ*	−2.7	−5.1
cRIG0368	**Malate dehydrogenase**	***mdh***	−10	−3.7
cRIG0847	**NAD(P) transhydrogenase subunit alpha**	***pntA1***	−2.8	−2.8
cRIG0313	Protein kinase C inhibitor 1	*pkcl*	−3.6	−6.6
cRIG0086	ABC-type transporter related to toluene tolerance	–	−9.5	−9.5
pRIG005	Transposase containing tnp_31 domain	*tnp*	^a^/0.9	^a^/3.2
**DISTINCT PATTERNS**				
cRIG0076	**Cell surface antigen Sca2**	***sca2***	+3.7	~1.9
cRIG0466	Folylpolyglutamate synthase	*folC*	+7.3	~1.8
cRIG0362	Lysyl-tRNA synthetase	–	+3.7	−4.1
cRIG0348	NADH dehydrogenase I chain C	*nuoC*	+3.7	−3.2
cRIG0864	**30S ribosomal protein S16**	***rpsP***	−3.6	~1.8
cRIG0307	Periplasmic protein TonB. links inner and outer membranes	*tonB*	−3.3	~1.5
cRIG0807	***Rickettsia*** **adhesin Adr1**	***adr1***	−3.8	~1.6
cRIG0578	Sco2 protein precursor	*sco2*	−4.2	~1.1
cRIG0014	**Cell surface antigen Sca1**	***sca1***	~1.3	+3.3
cRIG0869	Coproporphyrinogen III oxidase precursor	*hemF*	~1.5	+7.8
cRIG0380	Holliday junction DNA helicase RuvB	*ruvB*	~1.2	−9.5
cRIG0072	**OmpW family outer-membrane protein**	***ompW***	~1.9	−3.8
cRIG0284	VirB10 protein	*virB10*	~1.3	−3.2
cRIG0269	**Small heat shock protein**	***hsp1***	~1.4	−4.2
cRIG0621	**Nucleotide exchange factor protein GrpE**	***grpE***	~1.2	−7.1
cRIG0003	O-antigen export system ATP-binding protein	*rfbE*	~1.4	−3.5
cRIG0061	Deoxyguanosinetriphosphate triphosphohydrolase	*dgt*	~1.1	−7.4
cRIG0518	Dihydrodipicolinate synthase	*dapA*	^*^	−24.3

Bold and normal characters correspond to previously and newly identified proteins during rickettsial-host interactions, respectively. +, −, ~, ^a^/ or ^*^mean proteins either up-regulated, down-regulated, equally regulated, specific or un-classified, respectively.

## Discussion

Obligate intracellular bacterial pathogens are constantly evolving, in a bottleneck lifestyle, through various processes, including reductive evolution, selection, mutations, InDels, mobile genetic elements, etc. (Rohmer et al., 2007; Bryant et al., 2012). Members of the *Rickettsia* genus, which are obligate intracellular bacteria, exhibit diverse ecological and biological features that may involve multiple genes and genetic pathways during pathogen-host colonization and interactions that counteract host homeostasis and/or defense mechanisms. In this study, we performed comparative genomics, phylogenomics and proteomics on four SFG *Rickettsia* species in order to identify the genomic signature(s) and proteomic profiles, and to understand the evolutionary event(s), that distinguish *R. slovaca* and *R. conorii, two* of the virulent human rickettsial pathogens, from *R. raoultii* and *R. massiliae*, which cause the milder infections of two distinct diseases; i.e., SENLAT (Raoult and Roux, 1997; Parola et al., 2009; Foissac et al., 2013) and MSF (Cascio et al., 2013; Milhano et al., 2014; Bechelli et al., 2015; Portillo et al., 2015).

### Divergent and convergent evolution

Phylogenomic analysis revealed a divergent evolution of the virulent and the milder agents into two clades from a common ancestor. This divergence was corroborated by strong differences in structural variations (i.e., non-synonymous mutations and InDels) in the predicted core proteins (i.e., representing 71% of the pan-genome) between the virulent and milder agents. These data suggest that both evolutionary events might have contributed to the differences in virulence between the four SFG species. In a similar study, adaptive mutations were suggested to determine virulence in the highly pathogenic *R. prowazekii* (Zhang et al., 2006; Bechah et al., 2010). In another example, 115 non-synonymous SNPs were found between the virulent *Mycobacterium bovis* strains and the attenuated Bacillus Calmette-Guerin BCG strains, affecting important functions such as global regulators, transcriptional factors, and central metabolism, which may impact virulence (Garcia Pelayo et al., 2009). However, the phylogenomic analysis also showed that the virulent and milder agent causing SENLAT or MSF diseases are distantly related, suggesting that both agents of each rickettsiosis may have undergone discrete convergent evolution, as reported for similarities in intracellular strategies between phylogenetically distant microbes (Casadevall, 2008).

### Reductive evolution

The phylogenetic tree based on gene content dissimilarities reflected gene loss between the four taxa. Indeed, during and/or after their evolutionary divergence, the two virulent agents underwent more core gene losses and degradations, including the absence of any plasmid, as compared with the two milder species (three plasmids in *R. raoultii* and one in *R. massiliae*). Similar findings were observed in other species causing severe rickettsioses, including the TG *R. prowazekii* and *R. typhi*, which exhibit small chromosomes and are plasmidless (McLeod et al., 2004; Bechah et al., 2010; Clark et al., 2015), and other species causing mild or no disease, including the SFG *R. helvetica, R. africae, R. felis*, and *R. peacockii*, which have larger chromosomes and harbor one or more plasmids (Ogata et al., 2005; Felsheim et al., 2009; Fournier et al., 2009; Dong et al., 2012). In a recent study, we demonstrated that rickettsial plasmids have undergone a reductive evolution, similar to that observed in rickettsial chromosomes, possibly leading progressively to cryptic plasmids or complete plasmid loss (El Karkouri et al., 2016). Moreover, in the order *Rickettsiales*, no association was found between virulence and the acquisition of novel genes or the presence of plasmids (Darby et al., 2007). Overall, our data are consistent with the assumption that differences in virulence of the four SFG species in humans may result from reductive evolution (Parish et al., 2003; Parkhill et al., 2003; Moore et al., 2004; Lescot et al., 2008; Fournier et al., 2009; Merhej et al., 2014).

In this study, the core gene set present in the two virulent agents, but lost or altered in the two milder species as well as in other nonpathogenic and pathogenic *Rickettsia* species, did not include any previously described bacterial virulence factor, and only one core protein of unknown function exhibited a comprehensive protein abundance. This suggests that they cannot be linked to rickettsial pathogenesis and virulence in humans, or are unidentified virulence factors, and hence their roles need to be elucidated. However, the core gene set is conserved in the two milder agents and several other less pathogenic *Rickettsia* species, but deleted or in a degradation process in the two virulent species and other highly pathogenic *Rickettsia* species. Moreover, several of these core genes displayed comprehensive protein abundances, including for example those coding for glutaredoxin-like protein GrlA, multidrug resistance protein Atm1, 1-acyl-sn-glycerol-3-phosphate acyltransferase, patatin-like phospholipase Pat2, two transposases, a plasmid partitioning ParA family protein and a conserved protein of unknown function. Thus, the decay of the functions of these genes may contribute to the emergence of highly pathogenic bacteria, but their expressions in the milder agents may reflect antivirulence roles as demonstrated for example in *Shigella, Yersinia*, and *Francisella* antivirulence genes (Moore et al., 2004; Maurelli, 2007; Bliven and Maurelli, 2012), and/or may be related to host-adaptation, as was suggested for *Bordetella* genomes (Parkhill et al., 2003). None of these genes corresponded to any known antivirulence genes (e.g., *nad*A/B, *lac*I, *lpx*L, and *pep*O genes) identified in other bacterial pathogens (Bliven and Maurelli, 2012), suggesting that they may be unidentified or adaptive genes. Hence, the influence of these genes in increased virulence and/or in adaptation of the examined pathogenic phenotypes needs further functional analysis using, for example, genetic manipulation by the shuttle vector system developed from *R. amblyommii* plasmids or as demonstrated in *Burkholderia pseudomallei* and *Salmonella enterica* (Moore et al., 2004; Burkhardt et al., 2011; Bliven and Maurelli, 2012; Wood et al., 2012).

### The mobilome

It has been proposed that the proliferation of insertion of sequences (IS elements) is the cause of a large number of pseudogenes and genomic rearrangements in emerging or highly virulent pathogens (Parkhill et al., 2003; Wei et al., 2003; Petrosino et al., 2006; Rohmer et al., 2007). For example, in the facultative intracellular bacterium *F. tularensis*, IS elements and other evolutionary events were correlated with the emergence of strains pathogenic for humans (Rohmer et al., 2007). In contrast, an extraordinary proliferation of mobile genetic elements (≥650 transposases) contributed to a limited synteny in *R*. endosymbionts of *Ixodes scapularis*, an SFG species which is not known to invade vertebrate cells (Gillespie et al., 2012). Likewise, the non-pathogenic SFG *R. peacockii* showed an introduction and a proliferation in 42 copies of the ISRpe1 transposon (Felsheim et al., 2009). These evolutionary events were associated with extensive genome rearrangements and numerous deletions, including deletions of several genes (e.g., *ank, dsb*A, *rick*A, protease II, and *sca*1) and thought to be related to loss of virulence in this species (Felsheim et al., 2009). In the current study, the two milder *Rickettsia* species harbored more genes related to the mobilome, particularly transposases, integrases and phage sequences, than the virulent *Rickettsia* agents. This abundance of genes did not disrupt the putative virulence factors deleted in *R. peacockii*, suggesting that the mobilome in the milder species may have influenced their genomic stability with or without any impact on virulence in humans, or may improve their potential to gain novel genes of adaptation, survival and/or fitness. This study identified one integrase catalytic region and one phage-associated protein specific to the milder MSF agent, suggesting that the mobilome is still active. However, the lack of expression from the remaining genes of the mobilome suggests that they may be in a dormant or inactivated state.

### The pan-proteome is mainly coded by the core-genome

Remarkably, after infecting *X. laevis* cells, our study revealed that 96% of the pan-proteome was coded by 66% of genes from the core chromosome, whereas 4% of chromosomally-encoded proteins were identified in only up to three species, and 19% of genes in plasmids showed expressions. Although *Rickettsia* genomes evolved by reductive evolution, 1–2% of the genes, either split or fragments, may still be active, thus corroborating previous studies that demonstrated transcription of several split/fragment genes in *R. conorii* (Ogata et al., 2001), *Mycobacterium leprae* (Akama et al., 2009) and *Lactobacillus delbrueckii* (Zheng et al., 2016). The expressed split genes were thought to conserve functional domains, or proposed to function as a class of non-coding RNAs and act as riboregulators at both the transcriptional and post-transcriptional levels (Erdmann et al., 2001; Zheng et al., 2016). However, the remaining altered genes were not expressed, suggesting that they may have lost their functions (i.e., by becoming pseudogenes). In sum, the proportions of the identified proteins in the current study (40–52 and 17–37% from chromosomes and plasmids, resp.) were higher than those reported in other rickettsial species, including 12% in *R. felis* (Ogawa et al., 2007), 3 to 19% in *R. prowazekii* (Renesto et al., 2005; Tucker et al., 2011), and 2.6% in *R. conorii* (Zhao et al., 2016) using gel-based proteomics, but comparable to those of other intracellular bacteria, such as *M. tuberculosis* (41%) and *C. trachomatis* (Saka et al., 2011; Gopinath et al., 2015), using gel–free proteomics.

Examination of the pan-proteome in the four rickettsial agents detected several core genes (e.g., *omp*A/B, *rick*A, *pld, omp, tly*C, *ppc*E, *tlc*D1-3/5, and *stb*D) that had previously been associated with rickettsial adhesion to and invasion of host cells, motility, survival, and/or virulence (e.g., Blanc et al., 2005; Ellison et al., 2009; Sears et al., 2012; Qi et al., 2013; Rahman et al., 2013; Gong et al., 2014; Gillespie et al., 2015). However, these genes were found to be equally expressed or had un-classified expressions in *X. laevis* cells (data not shown), suggesting that these known virulence factors may be differentially expressed during the early stage of adhesion to and infection of *X. laevis*. Furthermore, some of them (e.g., *omp*A and/or *rick*A genes) were previously found either altered or lost, for example, in the pathogenic and non-motile *R. prowazekii* species and the pathogenic and motile *R. typhi*, as well as in the nonpathogenic *R. rickettsii* str Iowa and *R. peacockii* (Ogata et al., 2001; Ellison et al., 2008; Felsheim et al., 2009; Georgiades et al., 2011; Sears et al., 2012). In another study, the *pld* gene that was shown to be required for virulence of *R. prowazekii* displayed no defect in the avirulent *R. rickettsii* strain Iowa (Clark et al., 2015).

However, we cannot rule out the putative role of other known or unidentified proteins that may impact the differences in virulence and/or pathogenicity between the four SFG species. First, the two virulent agents were distinguished from the milder agents by two distinct clusters of protein abundances. Second, they exhibited less up-regulated than down-regulated proteins and nearly no specifically expressed proteins. Authors have reported that *Rickettsia* spp. survive by taking advantage of host cell nutrients (Andersson et al., 1998; Andersson and Andersson, 1999; Blanc et al., 2007a; Sahni and Rydkina, 2009). Thus, the current data suggest differences in intracellular strategies and biological activities which might reflect mechanisms of pathogenesis between the two virulent and two milder species.

In addition, the two virulent SENLAT and MSF agents displayed 72 proteins with similar patterns, in which 8 were up/up-regulated, 61 were down/down-regulated and three were plasmid-specific of the milder agents. These profiles may include putative virulence/antivirulence-associated proteins, which may have been influenced by the divergent driving forces revealed in this study. Indeed, evolutionary pressures may change or disrupt the structures of genes and their proteins, leading to differences in expressions and loss of function, respectively. In a proteomic study of the pathogenic *Shigella flexneri*, the down-regulated *arg*T gene was identified as an antivirulence gene which may interfere with the virulence factor of that species (Zhao et al., 2010). The similar patterns identified here mainly involved proteins associated with post-translational modification, protein turnover, chaperones (e.g., ClpX, TrxA, GroES, HtrA, and PrsA) and energy production and conversion (e.g., Mdh, FumC, SdhB, and Ppa), translation, ribosomal structure and biogenesis (e.g., RpmC, RplX′, DksA), cell wall/membrane/envelope biogenesis (e.g., Adr2, TolC and AmpD1) and general function prediction only (e.g., Pat1A, Pat2, and Ybgf). In contrast, the two virulent agents exhibited 268 proteins with distinct patterns. These profiles clearly discriminated the SENLAT agents from the MSF agents, and also the virulent from the milder agent of each rickettsiosis, suggesting that they may contain putative rickettsiosis-related proteins and/or specific virulence/antivirulence-related proteins within each disease. This may reflect convergent evolution related to rickettsial diseases and/or the divergent evolutionary history associated with a specific increase in virulence, respectively. Distinct patterns were found more in proteins related to translation, ribosomal structure and biogenesis (e.g., several tRNA synthetases), cell wall/membrane/envelope biogenesis (e.g., Adr1, LpxA, OmpW, Asma), energy production and conversion (e.g., NuoC/F/B/G, TlcD4, AtpX), replication, recombination and repair (e.g., recA/R, DnaB/N/E/Q/G), post-translational modification, protein turnover, chaperones (e.g., Hsp1/2, TrxB2, GroEL, ClpP, HslV) and general function prediction only (e.g., Uup succinate dehydrogenase iron-sulfur subunit and proteins with unknown functions), than in those involved in coenzyme transport and metabolism (e.g., HemA/B/F and FolC/D), intracellular trafficking, secretion, and vesicular transport (e.g., VirB61/3/4, VirB9-1/2, and VirB10) and amino acid transport and metabolism (e.g., DapF, PepE, IscS), and in lower numbers in proteins of the remaining categories.

## Conclusion

**Figure 8:**
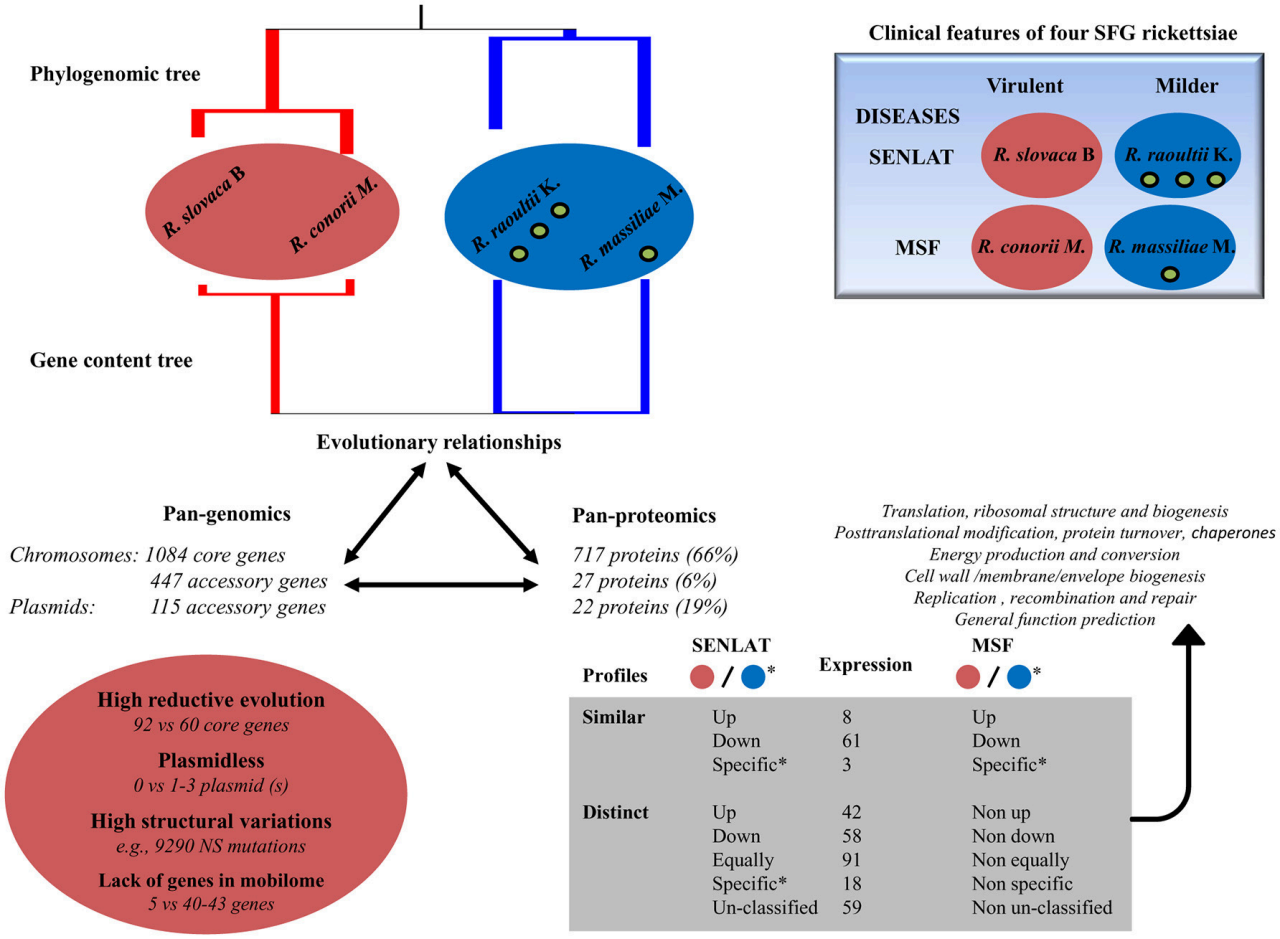
Summary of multi-omics results obtained from comparative analyses between four SFG rickettsiae including the virulent *R. slovaca* Rsl and the milder *R. raoultii* Rra which cause SENLAT diseases as well as the virulent *R. conorii* Rco and the milder *R. massiliae* Rma which cause MSF diseases. Up, Down, Equally, Specific and Un-classified mean up-regulated, down-regulated, equally regulated, specific and un-classified proteins, respectively. As an example, Non up means that the proteins can be either down-regulated, equally regulated, specific or un-classified proteins. Red and blue colors correspond to the most virulent and the milder agents, respectively. Overall, the two most virulent agents compared with the milder agents exhibited several driving forces that may be associated to differences in virulence and/or plasticity including divergent and reductive evolution, no plasmid, high structural variations and/or a lack of genes in the mobilome. The similarities in the disease (i.e., either SENLAT or MSF) between two distantly related species suggest a convergent evolution. Moreover, the virulent agents also displayed similar and distinct protein profiles mainly in six COG categories. These patterns may include putative virulence- and/or disease-associated proteins as well as putative antivirulence-related proteins of the milder agents.

In a bottleneck lifestyle associated with genetic drift, the four SFG *Rickettsia* species have been shaped by distinct evolutionary processes that may have strongly impacted gene conservation and protein profiles, mainly in those of the core genome, as summarized in Figure 8. Thus, these driving forces may have influenced intracellular strategies in these rickettsiae, and that may contribute to the emergence of distinct virulence and rickettsiosis in humans, distinguishing the virulent from the milder agents, and the SENLAT from the MSF diseases. Although the SFG lineage gathered several syntenic genomes, our study suggests that the mechanisms governing virulence and pathogenicity in the examined rickettsiae are more complex than imagined. Recent study has indicated that virulence in SFG rickettsiae is multifactorial (Clark et al., 2015). The current multi-omics data provide new insights into intracellular pathogen-host interactions, and suggest that *X. laevis* host-cells can be used a tool to clarify genetic determinants underlying rickettsial diseases. Further studies using animal models, small regulatory RNAs (Davids et al., 2002; Schroeder et al., 2015; Narra et al., 2016; Schroeder et al., 2016) and endothelial cell responses to rickettsial infections (Bechelli et al., 2015; Zhao et al., 2016) may improve understanding of the pathogenesis and fitness of the SFG rickettsiae, and intracellular pathogenic bacteria.

## Author contributions

DR, KE, and PF conceived the project. KE performed bioinformatic analysis: MK, NA, and SA performed experiments and wrote their corresponding material and methods. KE wrote a draft and edited the manuscript. KE, PF, and DR revised the manuscript.

### Conflict of interest statement

The authors declare that the research was conducted in the absence of any commercial or financial relationships that could be construed as a potential conflict of interest.
