# Supplementary material for: Multi-omics Analysis Sheds Light on the Evolution and the Intracellular Lifestyle Strategies of Spotted Fever Group Rickettsia spp
Source: Front Microbiol. 2017 Jul 20;8:1363. doi: 10.3389/fmicb.2017.01363 (PMC5517468; doi:10.3389/fmicb.2017.01363)
Supplement: Supplementary file 1 [file Image1.PDF]

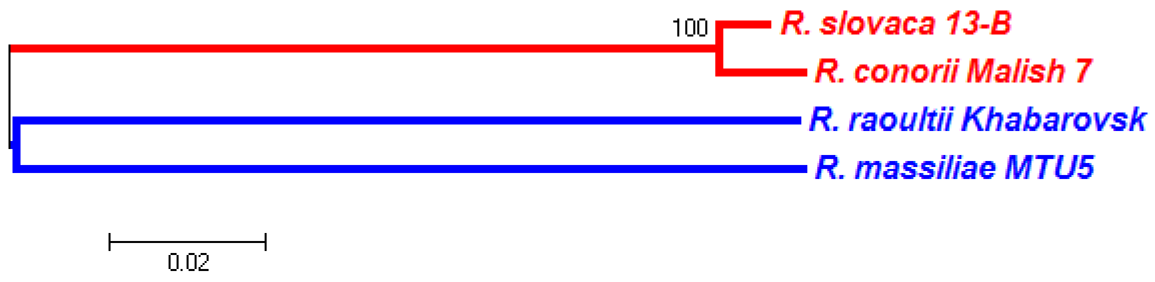

Figure S1. Neighbor-joining tree inferred from gene content dissimilarity values between the virulent agents *R. slovaca* Rsl and *R. conorii* Rco (red color) and the milder agents *R. raoultii* Rra and *R. massiliae* Rma (blue color). The distance or dissimilarity values were obtained using Jaccard's dissimilarity index and a binary matrix (coded 1 and 0 for presence and absence of a gene, resp.) extracted from pan-genome analysis (see Figure 2). Bootstrap supports higher than 90% are shown at the nodes.
