# Supplementary material for: Multi-omics Analysis Sheds Light on the Evolution and the Intracellular Lifestyle Strategies of Spotted Fever Group Rickettsia spp
Source: Front Microbiol. 2017 Jul 20;8:1363. doi: 10.3389/fmicb.2017.01363 (PMC5517468; doi:10.3389/fmicb.2017.01363)
Supplement: Supplementary file 2 [file Image2.PDF]

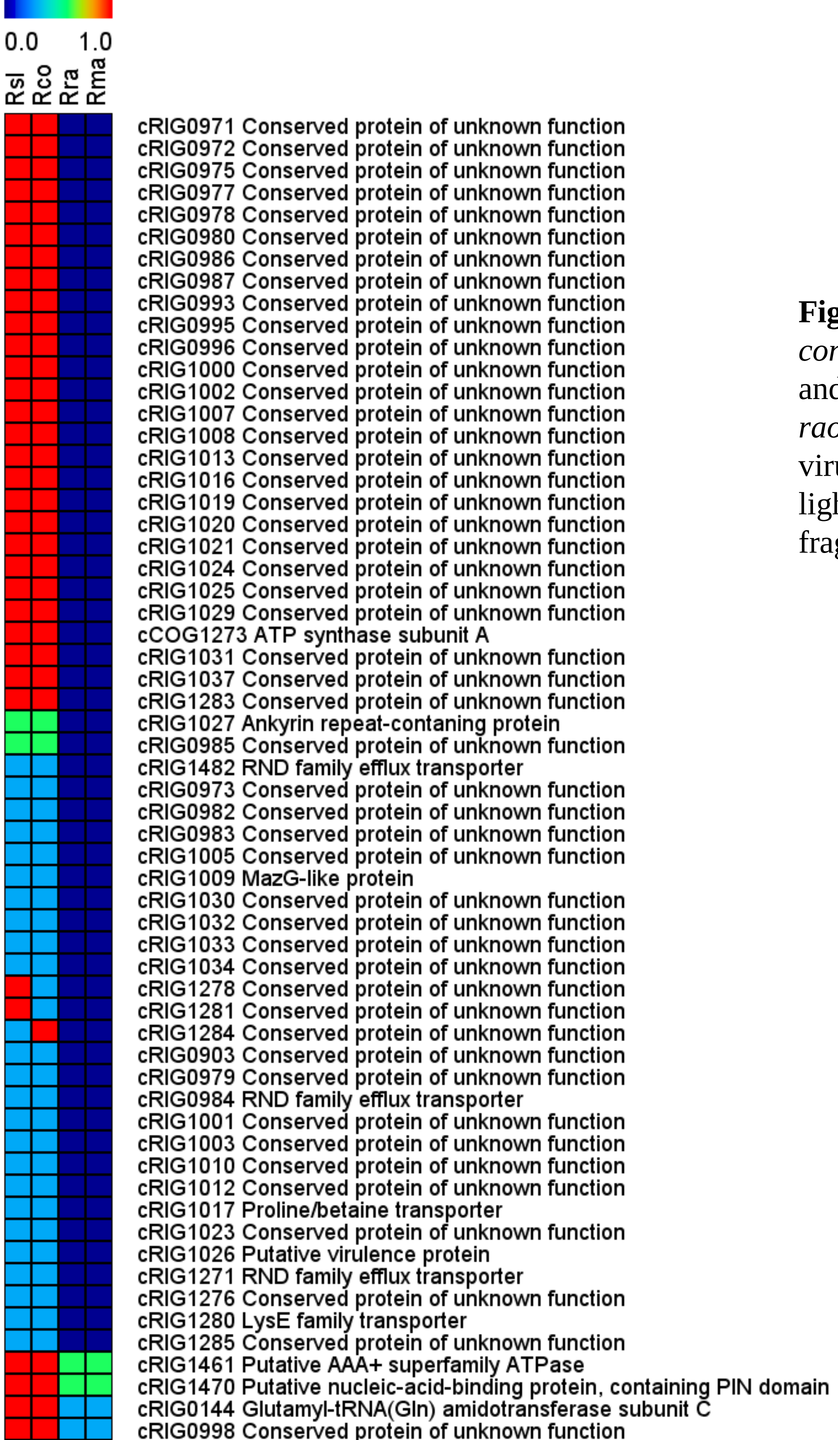

Figure S2. List of 60 genes of the virulent agents *R. slovaca* Rsl and *R. conorii* Rco that were lost or altered in the milder agents *R. raoultii* Rra and *R. massiliae* Rma (A), and 92 genes of the milder species *R. raoultii* Rra and *R. massiliae* Rma that were lost or altered in the virulent species *R. slovaca* Rsl and *R. conorii* Rco (B). Red, green, lightbleue and darkblue colours mean that genes can be complete, split, fragment and absent/remnant, respectively.

A

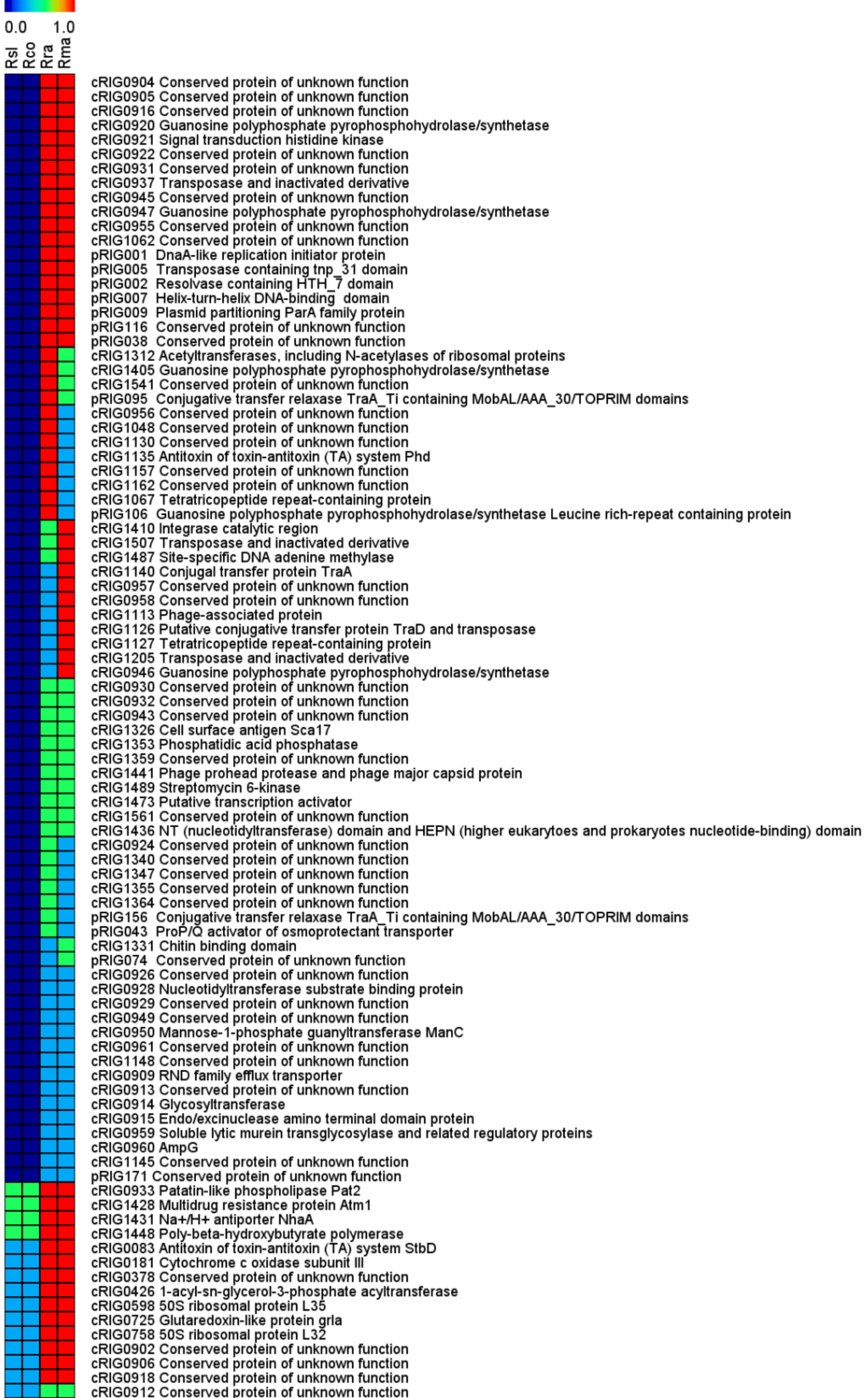
0.0
1.0
Rsl
Rco
Rra
Rma
cRIG0904 Conserved protein of unknown function
cRIG0905 Conserved protein of unknown function
cRIG0916 Conserved protein of unknown function
cRIG0920 Guanosine polyphosphate pyrophosphohydrolase/synthetase
cRIG0921 Signal transduction histidine kinase
cRIG0922 Conserved protein of unknown function
cRIG0931 Conserved protein of unknown function
cRIG0937 Transposase and inactivated derivative
cRIG0945 Conserved protein of unknown function
cRIG0947 Guanosine polyphosphate pyrophosphohydrolase/synthetase
cRIG0955 Conserved protein of unknown function
cRIG1062 Conserved protein of unknown function
pRIG001 DnaA-like replication initiator protein
pRIG005 Transposase containing tnp_31 domain
pRIG002 Resolvase containing HTH_7 domain
pRIG007 Helix-turn-helix DNA-binding domain
pRIG009 Plasmid partitioning ParA family protein
pRIG116 Conserved protein of unknown function
pRIG038 Conserved protein of unknown function
cRIG1312 Acetyltransferases, including N-acetylases of ribosomal proteins
cRIG1405 Guanosine polyphosphate pyrophosphohydrolase/synthetase
cRIG1541 Conserved protein of unknown function
pRIG095 Conjugative transfer relaxase TraA_Ti containing MobAL/AAA_30/TOPRIM domains
cRIG0956 Conserved protein of unknown function
cRIG1048 Conserved protein of unknown function
cRIG1130 Conserved protein of unknown function
cRIG1135 Antitoxin of toxin-antitoxin (TA) system Phd
cRIG1157 Conserved protein of unknown function
cRIG1162 Conserved protein of unknown function
cRIG1067 Tetratricopeptide repeat-containing protein
pRIG106 Guanosine polyphosphate pyrophosphohydrolase/synthetase Leucine rich-repeat containing protein
cRIG1410 Integrase catalytic region
cRIG1507 Transposase and inactivated derivative
cRIG1487 Site-specific DNA adenine methylase
cRIG1140 Conjugal transfer protein TraA
cRIG0957 Conserved protein of unknown function
cRIG0958 Conserved protein of unknown function
cRIG1113 Phage-associated protein
cRIG1126 Putative conjugative transfer protein TraD and transposase
cRIG1127 Tetratricopeptide repeat-containing protein
cRIG1205 Transposase and inactivated derivative
cRIG0946 Guanosine polyphosphate pyrophosphohydrolase/synthetase
cRIG0930 Conserved protein of unknown function
cRIG0932 Conserved protein of unknown function
cRIG0943 Conserved protein of unknown function
cRIG1326 Cell surface antigen Sca17
cRIG1353 Phosphatidic acid phosphatase
cRIG1359 Conserved protein of unknown function
cRIG1441 Phage prohead protease and phage major capsid protein
cRIG1489 Streptomycin 6-kinase
cRIG1473 Putative transcription activator
cRIG1561 Conserved protein of unknown function
cRIG1436 NT (nucleotidyltransferase) domain and HEPN (higher eukarytoes and prokaryotes nucleotide-binding) domain
cRIG0924 Conserved protein of unknown function
cRIG1340 Conserved protein of unknown function
cRIG1347 Conserved protein of unknown function
cRIG1355 Conserved protein of unknown function
cRIG1364 Conserved protein of unknown function
pRIG156 Conjugative transfer relaxase TraA_Ti containing MobAL/AAA_30/TOPRIM domains
pRIG043 ProP/Q activator of osmoprotectant transporter
cRIG1331 Chitin binding domain
pRIG074 Conserved protein of unknown function
cRIG0926 Conserved protein of unknown function
cRIG0928 Nucleotidyltransferase substrate binding protein
cRIG0929 Conserved protein of unknown function
cRIG0949 Conserved protein of unknown function
cRIG0950 Mannose-1-phosphate guanyltransferase ManC
cRIG0961 Conserved protein of unknown function
cRIG1148 Conserved protein of unknown function
cRIG0909 RND family efflux transporter
cRIG0913 Conserved protein of unknown function
cRIG0914 Glycosyltransferase
cRIG0915 Endo/excinuclease amino terminal domain protein
cRIG0959 Soluble lytic murein transglycosylase and related regulatory proteins
cRIG0960 AmpG
cRIG1145 Conserved protein of unknown function
pRIG171 Conserved protein of unknown function
cRIG0933 Patatin-like phospholipase Pat2
cRIG1428 Multidrug resistance protein Atm1
cRIG1431 Na+/H+ antiporter NhaA
cRIG1448 Poly-beta-hydroxybutyrate polymerase
cRIG0083 Antitoxin of toxin-antitoxin (TA) system StbD
cRIG0181 Cytochrome c oxidase subunit III
cRIG0378 Conserved protein of unknown function
cRIG0426 1-acyl-sn-glycerol-3-phosphate acyltransferase
cRIG0598 50S ribosomal protein L35
cRIG0725 Glutaredoxin-like protein grla
cRIG0758 50S ribosomal protein L32
cRIG0902 Conserved protein of unknown function
cRIG0906 Conserved protein of unknown function
cRIG0918 Conserved protein of unknown function
cRIG0912 Conserved protein of unknown function

B
