# Supplementary material for: Multi-omics Analysis Sheds Light on the Evolution and the Intracellular Lifestyle Strategies of Spotted Fever Group Rickettsia spp
Source: Front Microbiol. 2017 Jul 20;8:1363. doi: 10.3389/fmicb.2017.01363 (PMC5517468; doi:10.3389/fmicb.2017.01363)
Supplement: Supplementary file 3 [file Image3.PDF]

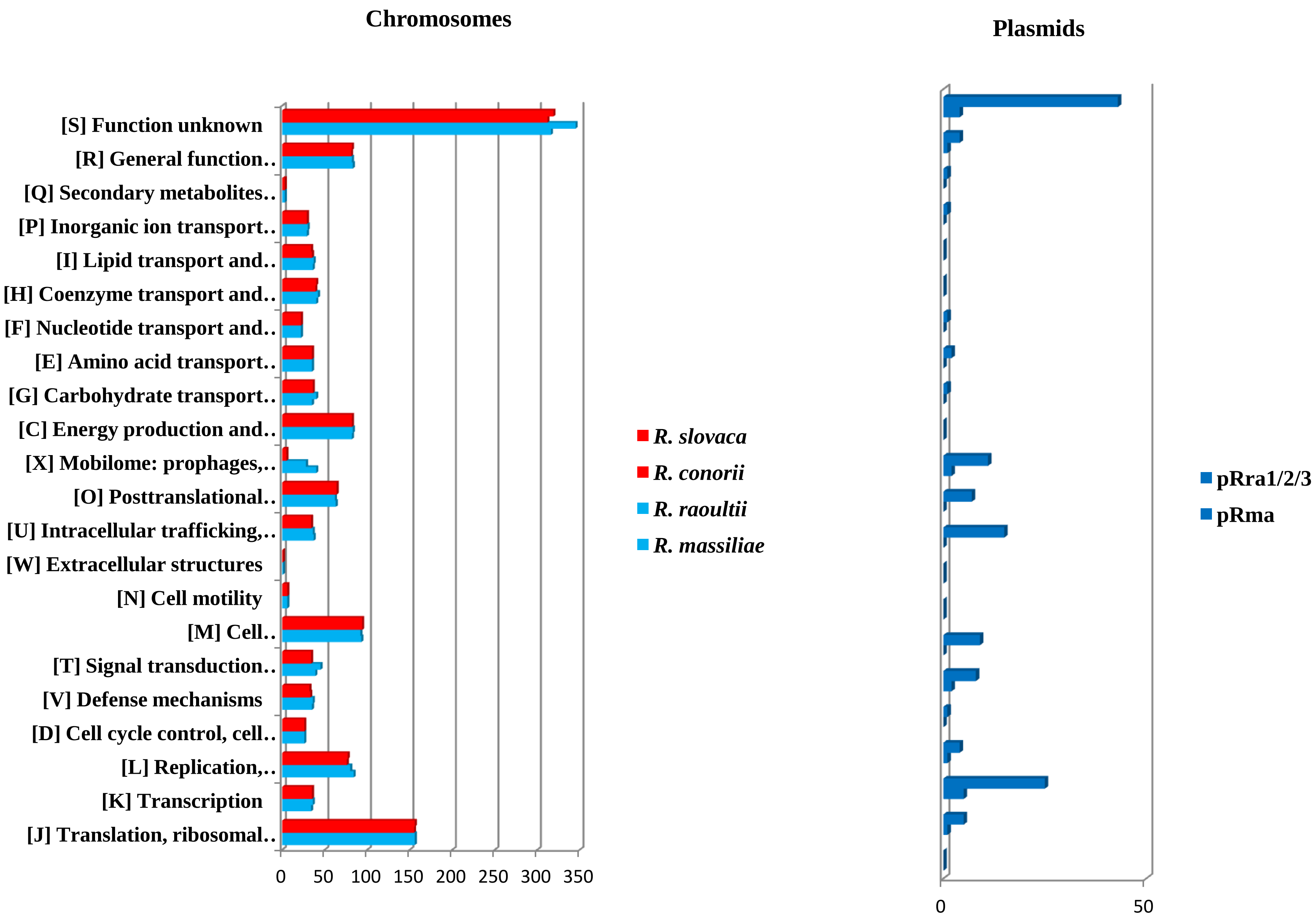

Figure S3. Comparative COG functional categories of gene contents between the virulent agents *R. slovaca* and *R. conorii*, and the milder agents *R. raoultii* and *R. massiliae*.
