# Supplementary material for: Multi-omics Analysis Sheds Light on the Evolution and the Intracellular Lifestyle Strategies of Spotted Fever Group Rickettsia spp
Source: Front Microbiol. 2017 Jul 20;8:1363. doi: 10.3389/fmicb.2017.01363 (PMC5517468; doi:10.3389/fmicb.2017.01363)
Supplement: Supplementary file 4 [file Image4.PDF]

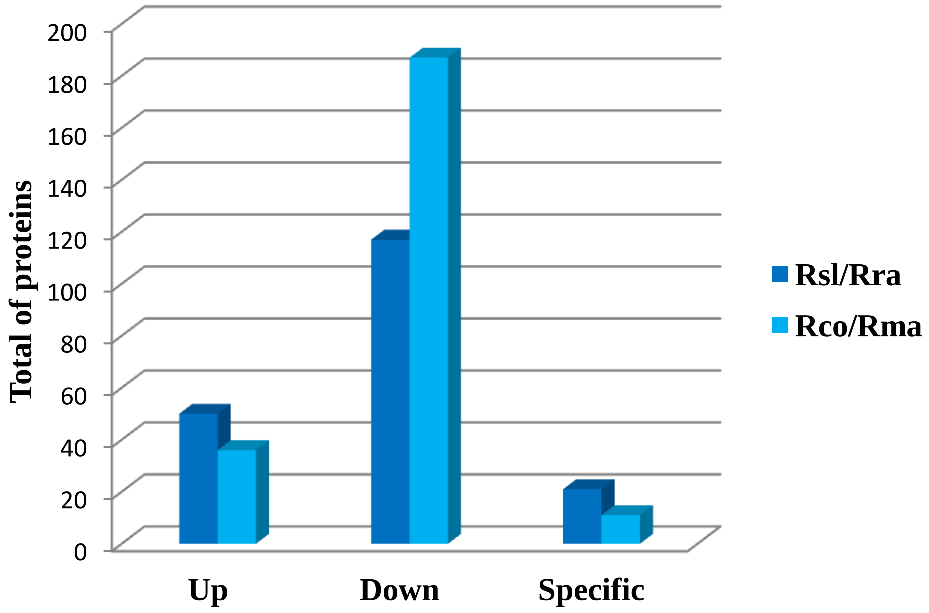

Figure S4. Protein profiles found between the SENLAT agents (the virulent *R. slovaca* Rsl and the milder *R. raoultii* Rra), and between the MSF agents (the virulent *R. conorii* Rco and the milder *R. massiliae* Rma). Up, Down and specific mean up-regulated, down-regulated and specific proteins.
