# Supplementary material for: Multi-omics Analysis Sheds Light on the Evolution and the Intracellular Lifestyle Strategies of Spotted Fever Group Rickettsia spp
Source: Front Microbiol. 2017 Jul 20;8:1363. doi: 10.3389/fmicb.2017.01363 (PMC5517468; doi:10.3389/fmicb.2017.01363)
Supplement: Supplementary file 5 [file Image5.PDF]

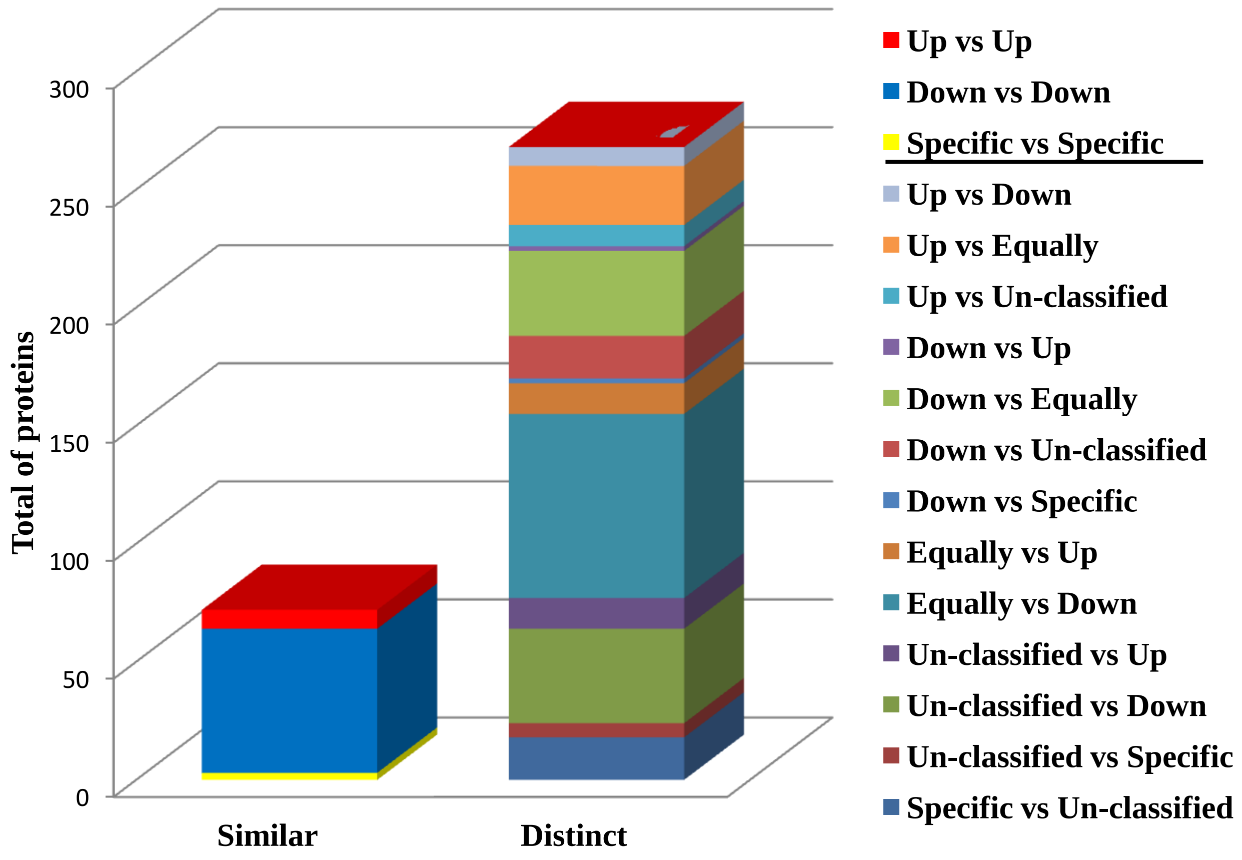

Figure S5. Similar and distinct protein patterns obtained between the SENLAT agents (the virulent *R. slovaca* Rsl / the milder *R. raoultii* Rra) and the MSF agents (the virulent *R. conorii* Rco / the milder *R. massiliae* Rma).
